# *Cyclocarya paliurus* Leaves Tea Improves Dyslipidemia in Diabetic Mice: A Lipidomics-Based Network Pharmacology Study

**DOI:** 10.3389/fphar.2018.00973

**Published:** 2018-08-28

**Authors:** Lixiang Zhai, Zi-wan Ning, Tao Huang, Bo Wen, Cheng-hui Liao, Cheng-yuan Lin, Ling Zhao, Hai-tao Xiao, Zhao-xiang Bian

**Affiliations:** ^1^School of Chinese Medicine, Hong Kong Baptist University, Kowloon, Hong Kong; ^2^School of Pharmaceutical Sciences, Health Science Center, Shenzhen University, Shenzhen, China; ^3^Shenzhen Research Institute and Continuing Education, Hong Kong Baptist University, Shenzhen, China

**Keywords:** *Cyclocarya paliurus*, *Diabetic dyslipidemia*, hyperlipidemia, lipidomic, network pharmacology

## Abstract

Hyperlipidemia and hepatic steatosis afflict over 75% of patients with type 2 diabetes, causing diabetic dyslipidemia. *Cyclocarya paliurus* (CP) leaf is a herbal tea which has long been consumed by the Chinese population, particularly people suffering from obesity and diabetes. CP appears to exhibit a hypolipidemic effect in lipid loaded mice (Kurihara et al., 2003), although the detailed mechanisms and active ingredients for this hypolipidemic effect have not yet been answered. In this study, we investigated the beneficial effects of CP and predicted the mechanisms by utilizing lipidomics, serum-pharmacochemistry and network pharmacology approaches. Our results revealed that serum and hepatic levels of total triglyceride (TG), total cholesterol (T-CHO), low-density lipoproteins (LDL) and high-density lipoproteins (HDL), as well as 30 lipids including cholesterol ester (CE), diglyceride (DG), phosphatidylethanolamine (PE), phosphatidylcholine (PC), and sphingomyelin (SM) in CP-treated mice were improved in comparison with untreated diabetic mice. In parallel, 14 phytochemical compounds of CP were determined in mice serum after CP administration. Mechanistically, the network pharmacology analysis revealed the predicted targets of CP’s active ingredients ALOX12, APP, BCL2, CYP2C9, PTPN1 and linked lipidome targets PLD2, PLA2G(s), and PI3K(s) families could be responsible for the CP effects on diabetic dyslipidemia. In conclusion, this study revealed the beneficial effects of CP on diabetic dyslipidemia are achieved by reducing accumulation of hepatic lipid droplets and regulating circulatory lipids in diabetic mice, possibly through PI3K signaling and MAPK signaling pathways.

**GRAPHICAL ABSTRACT:**
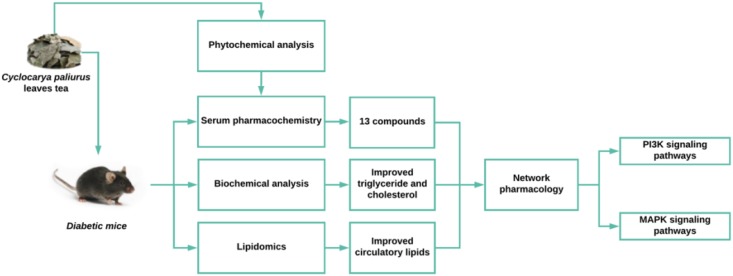
Work flow of the evaluation of the effects and mechanisms of *Cyclocarya paliurus* leaves tea on dyslipidemia in diabetic mice.

Work flow of the evaluation of the effects and mechanisms of *Cyclocarya paliurus* leaves tea on dyslipidemia in diabetic mice.

## Introduction

Hyperlipidemia and hepatic steatosis are frequently found in the metabolic syndrome and type 2 diabetes (Jung and Choi, 2014; Perry et al., 2014). Hyperlipidemia is characterized as high T-CHO, TG, LDL, and low HDL levels (Richhariya et al., 2015), whilst hepatic steatosis is represented by high TG, T-CHO, AST, and ALT levels (Stern and Castera, 2017). Emerging evidence suggest that dyslipidemia is a significant risk factor for the development of type 2 diabetes (Andriankaja and Joshipura, 2014; Association, 2015), and pharmacological lipid-lowering therapy is effective to alleviate the complications of type 2 diabetes including hyperlipidemia, hepatic steatosis, coronary heart disease, etc. (Zafrir and Jain, 2014; Sattar et al., 2016; Spence et al., 2016). *Cyclocarya paliurus* (Batal.) Iljinskaja (Juglandaceae) is a native medicinal plant grown in the southwest of China. The leaves of *C. paliurus* (CP) have been used as a herbal tea in China for its special flavor and the benefits to the obese and diabetic Chinese populations. The bioactive components isolated from CP are flavonoids, triterpenoids, organic acids, and polysaccharides, these components contribute to its versatile biological properties including antihyperglycemia, antihyperlipidemia, and antihypertension (Jiang et al., 2015; Yoshitomi et al., 2017). Previous studies have investigated the anti-diabetic function and mechanism of CP leaves on STZ and high-fat diet-induced type 2 diabetic mice, and its potent hypoglycemic effect has been verified on diabetic mice (Wang Q. et al., 2013; Ma et al., 2015). Moreover, CP has also been reported with hypolipidemic effects *in vivo* (Yao et al., 2015; Lin et al., 2016; Yang et al., 2016). However, the mechanism of bioactive constituents of CP on diabetic dyslipidemia remain unknown. Therefore, our objective is to study the effects of CP on lipid disorders in diabetes and elucidate its mechanism-of-actions.

It is acknowlegded that the majority of herbal medicines, including TCMs are orally delivered drugs of polypharmacy. Their active components are firstly absorbed into the bloodstream and then selectively and simultaneously interact with multiple targets at the root causes of the disease (Zhao et al., 2015). Serum pharmacochemistry is a rapid and reliable method using the UPLC-MS technique to track the components absorbed into the bloodstream and has been widely used to reveal the efficacy of TCMs (Yan et al., 2017). Moerover, metabolomics is an approach to analyze the metabolites, the intermediate products of metabolic reactions of living systems. This technology contains many subclasses based on the chemical characteristics of metabolites, namely lipidomics, amino metabolomics, and sugar metabolomics, etc. It has been widely used in the evaluation of the therapeutic effects and elucidation of the therapeutic mechanisms of herbal products (Wang X. et al., 2017). Metabolomics profiling can reveal whole metabolic profile changes of living systems in response to endogenous or exogenous stimuli such as drug treatment (Beger et al., 2016). Because any effects of herbal products are mediated by their constituents in a complex biological system, metabolomics can help us to analyze their action network comprehensively. Network pharmacology is a bioinformatics strategy to understand drug action and mechanisms by mapping drug-target-disease networks from the biological level (Li et al., 2014). To date, accumulating evidence suggest that network pharmacology approach is a powerful tool to study the molecular mechanisms of the complex components found in medicinal herbs.

Considering CP as a herbal tea with multiple components and diabetic dyslipidemia as a cluster of lipid abnormalities, we focused on the investigation of the beneficial effect of CP and its mechanisms against diabetic dyslipidemia using lipidomics, serum pharmacochemistry, and network pharmacology approaches.

## Materials and Methods

### Chemicals and Materials

#### Regents and Standards

UPLC grade organic solvent was purchased from Merck (Darmstadt, Germany). Deionized water was obtained from a milli-Q system (Millipore, Billerica, MA, United States). Formic acid, ammonium formate, and phosphoric acid were obtained from Sigma (St. Louis, MO, United States). Lipids standards including a lipids mixture of TG, CE, DG, PE, PC, and SM, etc, were purchased from Avanti Polar Lipid (Alabaster, AL, United States). Strptomycin and glibenclamide was purchased from Sigma (St. Louis, MO, United States).

#### Plant Material Preparation

The leaves of *Cyclocarya paliurus* (Batal.) Iljinskaja were collected and authenticated by Prof. Hu-Biao Chen from School of Chinese Medicine, Hong Kong Baptist University, and voucher specimens (No. CP20151201) was stored in our Research Laboratory. For the preparation of the extract of *C. paliurus* leaves (CP extract), the dried leaves of *C. paliurus* (5 kg) were soaked in boiled water (1:10 *m/v*) for 2 h twice. The solution was concentrated and dried under vacuum freezer to obtain crude extract. The crude extract was then extracted by 70% EtOH for 2 h (1:10 m/v) in an ultrasound bath. The refined solution was concentrated and dried under vacuum freezer again and the refined extract was stored in 4°C fridge until use. CP solution was prepared with 0.5% sodium carboxymethyl cellulose (CMC-Na) solution for animal oral administration and MeOH for phytochemical analysis.

### Animal Studies

#### Animal Handling and Diets

Eight-week-old C57/BL6J mice were purchased from Laboratory Animal Services Centre, Chinese University of Hong Kong and raised in the Laboratory Animal Services Center, School of Chinese Medicine, Hong Kong Baptist University. The mice were raised in a 12 h light/dark cycle, temperature controlled (22°C) standard animal room provided with diet and water *ad libitum.* All experimental protocols were approved by the Animal Ethics Committees of Hong Kong Baptist University, in accordance with “Institutional Guidelines and Animal Ordinance” (Department of Health, Hong Kong Special Administrative Region) (Registration No. LIUYE/15-16/01-CLNC). Body weight, food consumption, and blood glucose level were monitored each week. Blood glucose level was determined by OMRON glucometer (Beijing, China) using blood samples collected from the tail vein.

#### Animal Groups

The diabetes mice model was induced by high-fat diet (adjusted Calories Diet, 42% from fat) (No. 881372) (Harlan Laboratories, Inc., Indianapolis, IN, United States) for 4 weeks and intraperitoneal (*i.p*) injection with STZ (25 mg/kg) three times in following 3 days. The mice with fasting glucose level higher than 11 mmol/L were considered as diabetic mice, and the diabetic mice with consecutive 7-day hyperglycemia (11 mmol/L or greater) were used for the experiment. An equal volume of vehicle was injected into the control mice. The diabetic mice were then divided into the diabetic group (model group), the CP treatment group (CP group) and glibenclamide treatment group (positive group). For the CP treatment group, the mice were orally administrated 2 g/kg/day CP until end of the experiment according to previous experimental data (**Supplementary Figure S1**). The normal mice were divided into two groups: vehicle control group (0.5% CMC-Na solution treated, named as blank group) and CP-treated control group (CP treated, named as control group). CP-treated control group mice were orally administered 2 g/kg/day CP extract and blank group mice were orally administered same volume 0.5% CMC-Na solution as CP solution. Glibenclamide was given at 15 mg/kg/day in 0.5% CNC-Na solution to mice in positive group according to the previous study (Xiao et al., 2017).

#### Blood Sample Collection and Preparation

For serum pharmacochemistry study, mice were orally administered CP solution and anesthesia by 3% chloral hydrate through intraperitoneal injection after 10 mins. About 1 mL blood was collected in heparin-tube. Plasma was obtained after 3,000 rpm centrifuge for 30 min at room temperature. A total of 900 μL MeOH was added to 100 μL plasma and centrifuged at 14,000 for 10 min at 4°C to precipitate protein. A total of 800 μL supernatant was dried under vacuum concentrator within 30 min and dissolved in 200 μL 70% methanol for LC-MS analysis.

For lipidomics study, about 1 mL blood was collected from mice under anesthesia, serum was obtained after centrifuging at 3,000 rpm for 30 min at room temperature. A total of 50 μL serum was used for lipidomics study. Briefly, 250 μL Folch solvent with the internal standard was added in 50 μL serum and vortexed vigorously. Two phases were formed after centrifugation at 5,000 rpm for 15 min. The bottom layer was dried under vacuum concentrator and dissolved in 200 μL of ACN/IPA/H2O (65:30:5 v/v/v) for analysis.

Another 100 μL serum was used for clinical index analysis including ALT, AST, ALP, CREA and BUN using a biochemical analyzer (Hitachi 902 Automatic Analyzer; Hitachi, Japan). TG, TCHO, LDL, and HDL were analyzed using assay kits purchased from Nanjing Jiancheng Bioengineering Institute (Nanjing, China) according to the manufacturers’ instructions.

#### Hepatic Histopathology Analysis and Biochemical Analysis

At the end of the study, the mice were sacrificed, and liver tissue was collected for H&E staining to analyses histology changes. Liver tissue from sacrificed mice was soaked in 10% formalin solution (prepared by 1× phosphate-buffered saline, PBS) and fixed for 12 h. Samples were then made into paraffin sections as specimens and stained with hematoxylin and eosin (H&E) according to manufacturer’s protocol (Mayer’s Hematoxylin Solution, Sigma-Aldrich). The sections were observed and captured under microscopy. About other 50 mg liver tissues were homogenized in iced 1× PBS solution (1:10, *m/v*). The homogenized solution was used for TG and TCHO analysis following the protocol of Nanjing Jiancheng Bioengineering Institute (Nanjing, China).

### Phytochemical and Metabolomics Study

#### Phytochemical Analysis

The chromatographic analysis was performed on an Agilent 1290 UPLC system equipped with an autosampler, binary gradient pump, and PDA detector. The system was operated at 30°C and a water ACQUITY UPLC HSS T3 column (150 mm × 2.1 mm, 1.7 μm) was used. The injection volume was 5 μL and the mobile phase flow rate was 0.4 mL/min. Solvents that constituted the mobile phase were (A) 0.2% aqueous acetic acid and (B) acetonitrile. The elution conditions were as follows: 0–5 min, linear gradient 2–5% B; 5–10 min, linear gradient 5–10% B; 10–15 min, linear gradient 10–25% B; 15–25 min, linear gradient 25–40% B; 25–28 min, linear gradient 40–90% B. Peaks were detected at 254 nm. The compounds of CP extracts were characterized by retention time, MS/MS information and related chemical standards in positive and negative modes.

#### Lipidomics Study

The liquid chromatogram was performed on an Agilent 1290 UPLC system. A waters ACQUITY UPLC HSS C18 column (2.1 mm × 100 mm, 1.8 μm particle size) was used for separation. The column temperature was maintained at 40°C and autosampler temperature was maintained at 8°C. The separation was performed with mobile phase A and B within 20 min per sample. Phase A consists of 60:40 water/ACN in 10 mM ammonium formate and 0.1% formic acid, and phase B is made by 90:10 IPA/ACN with 10 mM ammonium formate and 0.1% formic acid. The linear gradient was as follow: 32% B (0–1.5 min); 32–45% B (1.5–4 min); 45–52% B (4–5 min); 52–58% B (5–8 min); 58–66% B (8–11 min); 66–70% B (11–14 min); 70–75% B (14–18 min); 75–97% B (18–21 min); maintain 97% B (21–25 min); decrease to 32% B (25–30 min); maintained at 32% B (Gregory et al., 2013). The injection volume is 2 μL for the positive mode and 6 μL for the negative mode.

XCMS package in R was used to convert chromatograms intensity into raw data for multivariate statistical analysis using metaboanalyst (Huan et al., 2017). For lipid identification, the frame *m/z* values were used to search information on LIPID MAPS, Human Metabolome Database (HMDB) and Metlin database. The matches were confirmed by lipids’ exact tandem mass (MS/MS) and retention time based on our internal standards (SPLASH^TM^ Lipidomix^®^ Mass Spec Standard, Avanti Polar Lipids, United States).

### Bioinformatics and Statistical Analysis

#### Potential Targets Prediction and Linking Analysis

The detected chemical components of CP in plasma after oral administration were imported into our in-house target prediction tool “MOST” (Huang et al., 2017) to construct the target prediction database. Targets from the prediction database were then used to determine the relevance with diabetic dyslipidemia with literature review, and the relevant genes were considered as CP targets for diabetic dyslipidemia. The lipids data were imported into Cytoscape to generate a metabolites-reaction-enzyme-gene database. The genes of significantly changed lipids in CP treatment group were considered as lipid targets of CP. The predicted targets and lipids targets of CP were combined together to upload to STRING (Szklarczyk et al., 2014) for PPIs analysis to link the action of predicted targets with lipids targets of CP.

#### Target-Diabetic Dyslipidemia Link Analysis

All determined and predicted targets were searched on PubMed using key words: [targets name] AND [diabetes] AND [lipidemia]. The targets which were studied on lipid disorders in diabetes were also included.

#### Statistical Analysis

GraphPad was used for statistical analysis of the biochemical data. Statistically significant differences (*p* < 0.05) in mean values were calculated by Student’s *t*-test or one-way ANOVA. The lipidomics data was processed by R package with XCMS. Metaboanalyst (Xia et al., 2015) was used for multivariate statistical analysis. Cytoscape was used trace associated gene-enzyme using KEGG database.

## Results

### CP Attenuated Diabetic Dyslipidemia in Diabetic Mice Induced by High Fat Diet and STZ

**FIGURE 1:**
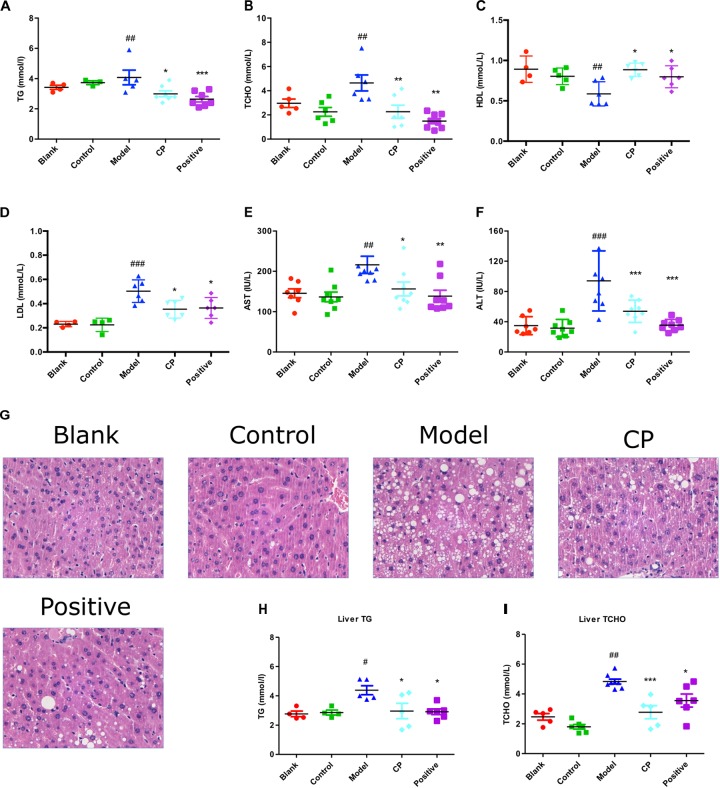
*Cyclocarya paliurus* attenuated diabetic dyslipidemia and hepatic steatosis induced by high fat diet and STZ in mice. Blank: non-diabetic control group; Control: CP-treated non-diabetic control group; Model: STZ and high fat diet-induced diabetic model group; CP: CP treated model group; Positive: glibenclamide treated model group. **(A–F)** Serum TG, TCHO, HDL, LDL, AST, and ALT level. **(G)** Histology analysis of liver tissues by **H,E** staining. **(H,I)** Liver TG and TCHO level. All data are presented as means + SEM (*n* = 6∼8). ^#^*p* < 0.05, ^##^*p* < 0.01, ^###^*p* < 0.001 (comparison between control and model group); ^∗^*p* < 0.05, ^∗∗^*p* < 0.01, ^∗∗∗^*p* < 0.001 (comparison between CP group, positive group, and model group).

As shown in **Table 1**, after high fat diet and STZ treatment, blood glucose levels of mice were significantly elevated whereas their body weight was significantly demoted compared with control and blank group, indicating that the diabetic mice model was well established. In contrast, those clinical signs were significantly rescued after CP treatment for 5 weeks. Since both hyperlipidemia and hepatic steatosis are characterized as substantial lipid disorders in diabetes, we determined serum TCHO, LDL, HDL, and TG levels, and hepatic AST, ALT, TG, and TCHO levels. Liver histopathological changes were also monitored. As shown in **Figure 1** and **Supplementary Figure S4**, compared to that of control and blank group, serum TG, TCHO, and LDL levels, as well as hepatic AST, ALT, TG, and TCHO levels of the model group were significantly increased, whereas serum HDL levels were significantly decreased, indicating that the hyperlipidemia and hepatic steatosis were developed in the model group. In CP or glibenclamide-treated groups, those altered lipids-associated indexes were significantly alleviated. In addition, histological examination of hepatic sections also revealed that there were a large number of vesicles of fat accumulating within hepatocytes of diabetic mice, which was significantly reduced after CP or glibenclamide treatment. These results suggested that CP extract has a great potential to attenuate diabetic dyslipidemia and hepatic steatosis induced by high fat diet and STZ in mice.

**Table 1:** Blood glucose (mM) levels and body weight changes on week 0, 3, and 5.

	Blank	Control	Model	CP	Positive
**Blood glucose (mM)**					
Week 0	6.8 ± 1.2	6.1 ± 0.9	17.0 ± 3.5^###^	16.2 ± 4.3	16.5 ± 5.2
Week 3	6.7 ± 1.0	6.6 ± 1.4	17.5 ± 4.0^###^	12.7 ± 3.2^∗^	12.6 ± 3.5^∗^
Week 5	6.0 ± 1.0	5.1 ± 0.5	16.6 ± 3.0^###^	10.7 ± 1.5^∗∗∗^	10.4 ± 2.0^∗∗∗^
**Body weight (g)**					
Week 0	23.8 ± 0.8	23.5 ± 1.8	24.4 ± 1.0	24.4 ± 1.0	24.5 ± 0.9
Week 3	24.6 ± 1.0	25.0 ± 1.0	23.4 ± 1.8^#^	24.1 ± 0.3	23.9 ± 1.3
Week 5	26.3 ± 1.2	26.2 ± 1.3	23.2 ± 1.8^##^	24.9 ± 0.6^∗^	24.6 ± 1.2^∗^


*The effects of CP extract on blood glucose levels and body weight of diabetic mice. All data are presented as means **±** SD. Diabetic model group vs. control group: ^#^p < 0.05, ^##^p < 0.01 and ^###^p < 0.001. CP or glibenclamide (positive) treatment group vs. diabetic model group: ^∗^p < 0.05, ^∗∗^p < 0.01, ^∗∗∗^p < 0.001.*

### CP Improved Circulating Lipid Metabolism in Diabetic Dyslipidemia

**FIGURE 2:**
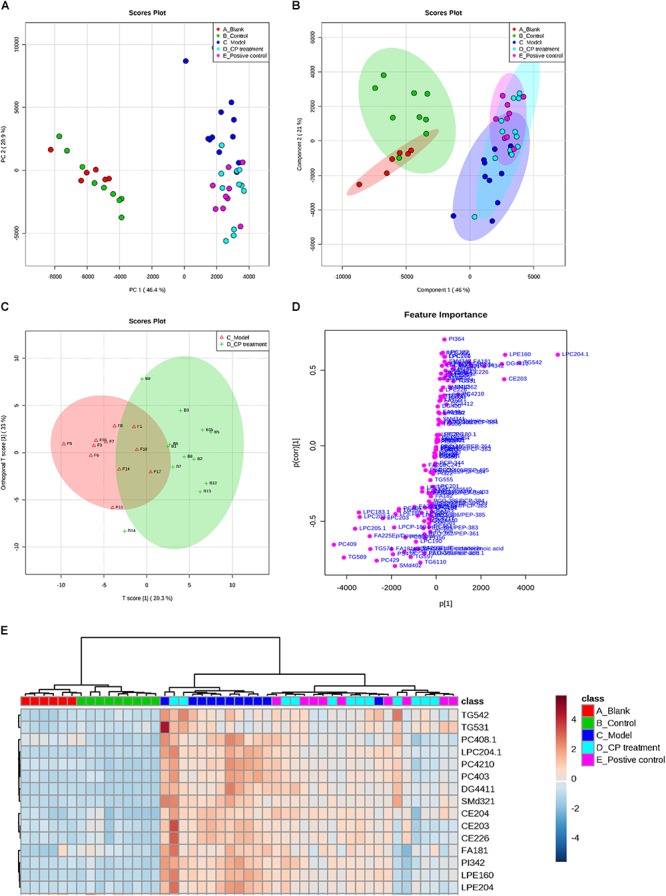
The lipidomics study on serum from diabetic mice: Metabolic profiles and multivariate data analysis. **(A)** PCA score plot of the blank group (red color), control group (green color), model group (dark blue), CP group (light blue), and positive group (purple). **(B)** PLS-DA score plot with 95% confidence interval of all studied groups. **(C,D)** OPLS-DA score plot and S-plot between model group and CP treatment group. **(E)** Heatmap of selected metabolites was built based on one-way ANOVA rankings and hierarchical clustering. Each dot represents one mouse.

To study the CP effect on lipid metabolism in diabetic mice, we performed a lipidomics study on mice serum. Fatty acyls (fatty acids), glycerolipids (DG and TG), glycerophospholipids (PC, PE, and PI), and sphingolipids (SM) were then used for multivariate statistical analysis as shown in **Supplementary Table S1.1**. As the PCA and PLS-DA are two effective multivariate analysis methods for large-scale data matrix, the combining data were analyzed using PCA and PLS-DA approaches. As shown in **Figures 2A,B**, PCA and PLS-DA score showed a clear classification between model group and control group, demonstrating that circulating lipids profiles are significantly different in the model group. As well, the CP-treated group or glibenclamide treated group also had its own distinctive classification, bearing some overlap. Considering the overlap between model group and CP-treated group, we further narrowed down the profiles of lipids in these two groups using OPLS-DA method. Notably, CE, DG, TG, PC, and SM were significantly distinguished between these two groups (**Figures 2C,D**), And then, we applied the hierarchal clustering analysis to segregate the lipid metabolites into three distinct groups (shown in **Figure 2E**). A significant number of lipids were up-regulated in model group in comparison with control or blank groups. After CP or glibenclamide treatment, the levels of these lipids were greatly down-regulated. The results suggest that CP or glibenclamide could significantly regulate lipid metabolism in diabetes. Interestingly, PCA and PLS-DA analysis result showed that the score plot of CP-treated group and glibenclamide-treated group were not separated, and the same result was observed in heat map of hierarchal clustering analysis (**Figures 2B,E**), suggesting CP and glibenclamide may share the similar metabolic pathways to improve the diabetic dyslipidemia.

**FIGURE 3:**
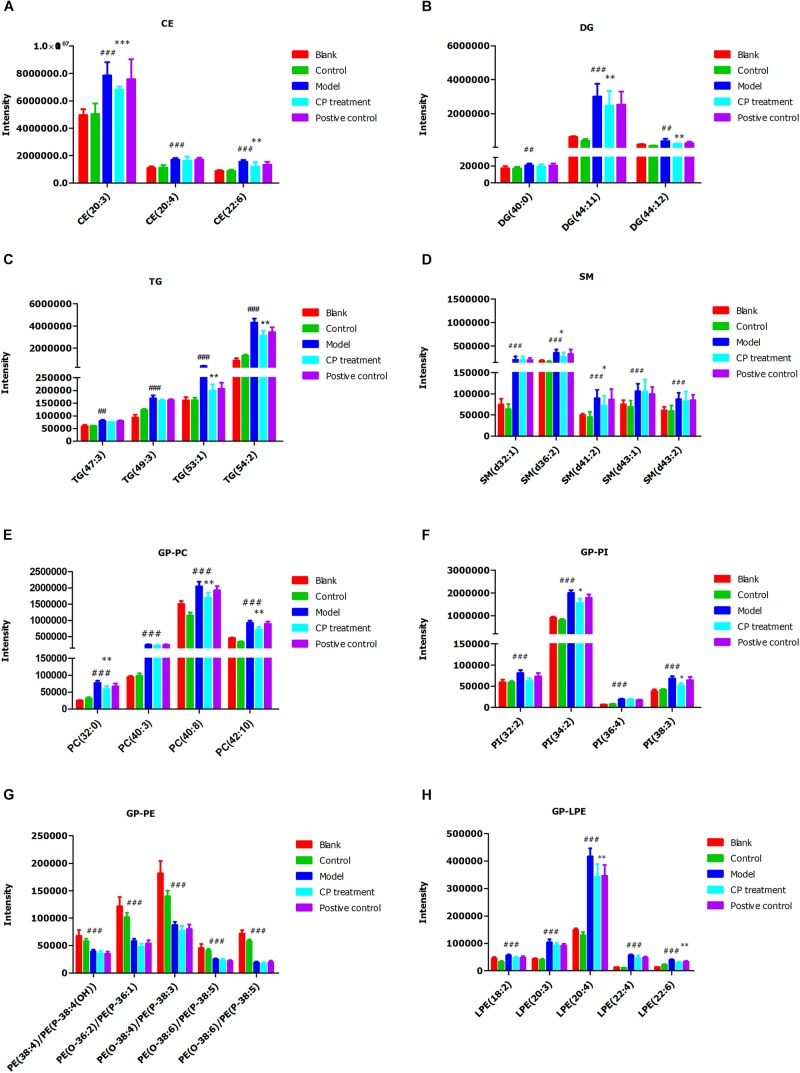
*Cyclocarya paliurus* improved circulating lipids profiles in diabetic mice model. **(A)** Cholesterol ester (CE), **(B)** Diglyceride (DG); **(C)** Triglyceride (TG); **(D)** Sphingomyelin (SM); **(E)** Phosphatidylcholines (GP-PC); **(F)** Phosphatidylinositol (PI); **(G)** Phosphatidylethanolamine (GP-PE); **(H)** Lysophosphatidylethanolamine (GP-LPE). All data are presented as means ± SEM (*n* = 6∼8). ^#^*p* < 0.05, ^##^*p* < 0.01, ^###^*p* < 0.001 (comparison between control and model group); ^∗^*p* < 0.05, ^∗∗^*p* < 0.01, ^∗∗∗^*p* < 0.001 (comparison between CP group and model group).

Subsequently, we analyzed the changes of lipids between control (blank) groups and model groups, and found that 47 lipids were elevated and 53 lipids were reduced in model group. After CP treatment, 30 lipids including CE, DG, TG, PC, and SM species were significantly either up-regulated or down-regulated (*p* < 0.05) (**Figure 3**). After importing those CP-regulated lipids to Cytoscape, a network pathway was built based on KEGG database and revealed that metabolic pathways including arachidonic acid metabolism, bile acid biosynthesis, *de novo* fatty acid biosynthesis, glycerophospholipid metabolism, glycosphingolipid metabolism, linoleate metabolism and saturated fatty acids beta-oxidation are involved in the regulating function of CP in lipid metabolism (As shown in **Supplementary Figure S5**).

### CP Possess Multiple Components-Multiple Targets-Multiple Pathways Properties for Diabetic Dyslipidemia

Since absorption into the bloodstream is one of the prerequisites for drug efficacy, we performed a pharmacochemistry study to detect the CP components in blood stream after oral administration in mice. As shown in **Table 2** and **Supplementary Figure S2**, after oral administration of CP to normal C57/BL6 mice, 13 compounds in total: quinic acid, neochlorogenic acid, chlorogenic acid, 4-hydroxybenzoic acid, gallic acid, quercetin-3-glucuronide, kaempferol, loganin 7-pentoside, astragalin, kaempferol-3-rhamnoside, quercetin, quadranoside IV, and asiatic acid were detected. Subsequently, we used in-house tools “MOST: most-similar ligand-based approach to target prediction” (Huang et al., 2017) to predict the protein targets of major potential active components of CP identified in bloodstream as shown in **Supplementary Table S1.2**. Sixty-nine targets were matched for flavonoids, 59 targets were matched for organic acids, and 2 targets were matched for saponins. There targets were searched by related key words online to determine the relevance of diabetic dyslipidemia. Five targets were selected after reference searching. Finally, we linked the lipids targets with predicted targets using STRING (Szklarczyk et al., 2014) to build interaction networks of predicted targets and lipids targets via PPI. Among these compounds, the main predicted targets of CP were ALOX12, APP, BCL2, CYP2C9, and PTPN1 while the predicted-targets linked lipids targets via PPI analysis were PLA2G(s) and PI3K(s) families as shown in **Supplementary Figure S3**.

**Table 2:** Constituents of CP identified in plasma.

No.	Compound	Classification	Blank plasma	Plasma after 10 min administration	Plasma after 30 min administration
1	Quinic acid	Organic acid	–	√	√
2	Neochlorogenic acid	Organic acid	–	√	–
3	Chlorogenic acid	Organic acid	–	√	√
4	4-Hydroxybenzoic acid	Organic acid	–	√	√
5	Gallic acid	Organic acid	–	√	–
6	Quercetin-3-glucuronide	Flavonoid	–	√	√
7	Astragalin	Flavonoid	–	√	√
8	Kaempferol	Flavonoid	–	√	√
9	Loganin 7-pentoside	Flavonoid	–	√	√
10	Kaempferol-3-rhamnoside	Flavonoid	–	√	√
11	Quercetin	Flavonoid	–	√	√
12	Quadranoside IV	Saponin	–	√	√
13	Asiatic acid	Saponin	–	√	√


**FIGURE 4:**
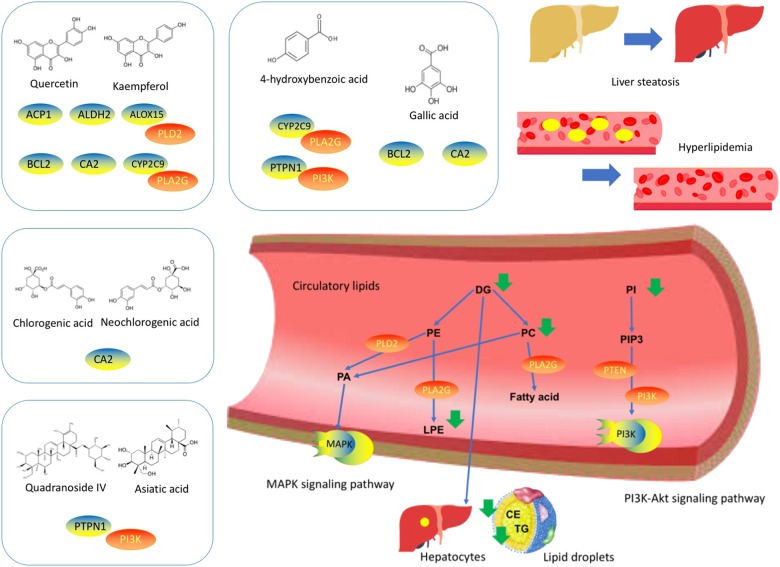
*Cyclocarya paliurus* possess multiple components-multiple targets-multiple pathways properties for diabetic dyslipidemia. **Left** to **right:** The predicted targets based on CP constituents. The PPI network of predicted targets-linked lipid targets. The lipids regulated by CP in diabetic dyslipidemia.

We then conducted reference searching to review the experiment study on these targets as shown in **Supplementary Table S2**. The ALOX, BCL-2, CYP 2C9, PLA2G(s) and PI3K(s) families, PLD2 and PTEN were determined as quercetin and kaempferol targets on diabetic dyslipidemia (Guo et al., 2017). PTPN1, PI3K(s) family and PLD2 were determined as targets of saponins such as quadranoside IV and asiatic acid (Ramachandran and Saravanan, 2015), whilst BCL-2, PI3K(s) family, PLD2 and PTEN were determined as action targets of gallic acid and 4-hydrobenzoic acids (**Figure 4**).

## Discussion

Hyperlipidemia is the most common form of dyslipidemia, refers to the abnormally elevated levels of any/all lipids or lipoproteins in the blood, frequently happened in long-term type II diabetes patients (Dixit et al., 2014). Recent studies have indicated that diabetic dyslipidemia may not only be the consequence but also the cause of disturbed glucose metabolism (Parhofer, 2015). Recently, CP was reported to improve insulin sensitivity (Jiang et al., 2014), attenuates inflammation (Wang Z. et al., 2017), and control hyperglycemic and hyperlipidemic abnormalities (Xu et al., 2017) both *in vitro* and *in vivo*, although the hypolipidemic mechanism has not been elucidated yet. In this study, we confirmed CP alleviated lipid dysfunction in diabetes, particularly diabetic dyslipidemia, as revealed by the clinical index, histological analysis, and lipidomics analysis. Mechanistically, we employed a network pharmacology approach to determine that CP’s hypolipidemic effect involvement in PI3K and MAPK signaling pathways.

Lipidomics is a powerful tool to investigate lipid profiles changes. Previous studies indicated that circulating lipidomes were correlated with hyperlipidemia and hepatic steatosis (Wouters et al., 2008), therefore making lipids profiles an adequate indicator of CP effects in this study. Through the lipidomics study, it was shown that STZ and high-fat diet induced type 2 diabetic mice had lipid disorders due to lipid metabolism changes. Among the changed lipids, the majority lipids classes were DG, TG, CE, PC, SM, FA, PI, and PE. We found that CE, DG, TG, SM, PC, PI, and LPE were increased in the model group, while PE was decreased in the model group. CE is a dietary lipid involved in fat digestion and absorption as well as cholesterol metabolism. It directly affects LDL as well as HDL levels, and can be hydrolyzed by pancreatic enzymes to produce cholesterol and free fatty acids (Rosenson et al., 2016). DG and TG represent the main lipid components of fat deposits, which accumulated largely in lipid droplets of hepatocytes when in hepatic steatosis (Szczepaniak et al., 2005). Our results showed that CE, DG, and TG were downregulated after CP treatment which further indiacates the reduxction in fat accumulation in lipid droplets. PC (lecithin) are one class of glycerophospholipids with choline as a head group, which constitute major component of biological membranes, associating with the health benefits such as liver repair and lipolysis (Payne et al., 2014). Lipid absorption and utilization, particularly TG, is closely related to the PC content and its associated enzymes. Besides, PC can protect against steatosis in mice (Niebergall et al., 2011). Our results displayed that CP could reduce the palmitic acids levels and adjust PC content to improve lipid utilization and protect hepatic steatosis. PE is directly associated with diabetes and dyslipidemia and PC/PE ratio is closely correlated with the accumulation of hepatic TG content (Bradley et al., 2015; Ling et al., 2017). Our results showed that the levels of PE were elevated in CP-treated diabetic mice, indicating CP can reduce TG accumulation. SM contains either PC or PE as its head group. As lipid rafts, SM takes part in lipid microdomains and inhibition of SM synthesis would increase the ceramide level, a mediator of non-alcoholic fatty liver diseases (Kasumov et al., 2015). Our results showed that PC could increase SM content by reducing ceramide levels, possibly to alleviate hepatic steatosis. Our results indicate that CP has a great potential in the improvement of circulating lipid metabolism in diabetic dyslipidemia.

The beneficial function of CP on diabetic dyslipidemia can be deduced by its lipid regulating effect on circulatory system through multiple metabolic pathways. Therefore, it is important to identify the CP components and elucidate their biological activities to further define its use as a herbal product. Moreover, identifying the targets of CP’s active components can lead to greater understanding of CP’s mechanisms of action against diabetic dyslipidemia. By using lipidomics-based network pharmacology approach, we determined several CP targets for diabetic dyslipidemia based on its potential active components. Besides of ACP1, ALDH2, BCL2, and CA2 targets, the ALOX15, CYP2C9, and PTPN1 targets can interact with lipid targets PLD2, PTEN, PLA2G(s), and PI3K(s) family targets. BCL2, PTPN1, and PI3K alters expression of PI3K signaling pathway (Rahmani et al., 2013; Sugiyama et al., 2017). ALDH2, ALOX15, and BCL2 are involved in MAPK signaling pathway (De Chiara et al., 2006; Zhang et al., 2011; Zhao et al., 2011), and activation of MAPK can influence the expression of CYP2C9 (Bachleda et al., 2009). PLD2, and its product phosphatidic acid, can also activate MAPK (Grab et al., 2004). Therefore, the signaling pathway for its pharmacological mechanism may be related with PI3K and MAPK signaling pathway (Ma et al., 2015; Wu et al., 2017; Xiao et al., 2017). The multiple components of CP targeting multiple targets through multiple metabolic pathways to improve diabetic dyslipidemia in type 2 diabetic mice.

To our knowledge, we have provided a novel metabolomics-based network pharmacology approach to combine and link experimental targets and predicted targets together based on the bioactive components of herbal products. For a network pharmacology study, particularly on herbal medicine or herbal formula, the metabolomics-based network pharmacology strategy can integrate more comprehensive targets for targets prediction and linking their interactions. Integrating the analysis of systemic metabolic profiles based on metabolomics study with computational prediction based on serum pharmacochemistry information can result in more precise pharmacological mechanism associated targets prediction (Huang et al., 2018). It is also the first study to link lipids targets from lipidomics study with predicted targets from computational tools through PPI to improve the network pharmacology analysis. Up to now, there are some questions that still need to be answered: First, some of PPI between predicted targets and lipid targets have not been validated experimentally. Further experimental work can be performed to uncover the relationship between targets-targets. Next, the potential active compounds we identified in this study have been shown to be effective for anti-diabetic studies on animals, the systemic evaluation of combinatorial use on these compounds have not been conducted yet. Additional *in vitro* and *in vivo* studies in future will help to uncover the multiple pharmacological mechanisms found in herbal medicines.

## Conclusion

In this study, we report CP attenuated diabetic dyslipidemia and hepatic streatosis in high fat diet and STZ-induced diabetic mice. The lipidomics study revealed CP improves circulatory lipids disorder, and the serum pharmacochemistry study revealed quinic acid, neochlorogenic acid, chlorogenic acid, 4-hydroxybenzoic acid, gallic acid, quercetin-3-glucuronide, kaempferol, loganin 7-pentoside, astragalin, kaempferol-3-rhamnoside, quercetin, quadranoside IV, and asiatic acid are potential active components of CP. Combining lipidomics and bioinformatics analysis, ALOX12, APP, BCL2, CYP2C9, and PTPN1 were predicted as direct targets of CP, whilst PLD2, PTEN and PLA2G(s) and PI3K(s) families were predicted as lipids linked targets of CP in diabetic hyperlipidemia. In conclusion, the CP was shown to be a multi-component and multi-targets herbal product with potent lipid regulation properties in dyslipidemia.

## Author Contributions

Z-xB and H-tX designed the study and revised the manuscript. Ht-X, BW, C-hL, LxZ, and Z-wN performed the animal experiment. LxZ performed the clinical index analysis, lipidomics analysis, bioinformatics analysis, and wrote the manuscript. Z-wN performed phytochemicals analysis of CP. TH performed bioinformatics analysis and revised the manuscript. LZ and C-yL provided technical support and advices toward study.

## Conflict of Interest Statement

The authors declare that the research was conducted in the absence of any commercial or financial relationships that could be construed as a potential conflict of interest.

## Abbreviations

ALP: alkaline phosphatase
ALT: alanine aminotransferase
AST: aspartate aminotransferase
BUN: urea nitrogen
CE: cholesterol ester
CP: *Cyclocarya paliurus*
CREA: creatinine
DG: diglyceride
HDL: high-density lipoproteins
LDL: low-density lipoproteins
PC: phosphatidylcholine
PE: phosphatidylethanolamine
PPI: protein–protein interaction
SM: sphingomyelin
STZ: streptozotocin
T-CHO: total cholesterol
TG: total triglyceride

## References

[B1] Andriankaja O. M. Joshipura K. (2014). Potential association between prediabetic conditions and gingival and/or periodontal inflammation. *J. Diabetes Investig.* 5 108–114. 10.1111/jdi.12122 24729853 PMC3980950

[B2] Association A. D. (2015). Standards of medical care in diabetes—2015 abridged for primary care providers. *Clin. Diabetes* 33 97–111. 10.2337/diaclin.33.2.97 25897193 PMC4398006

[B3] Bachleda P. Vrzal R. Dvorák Z. (2009). Activation of MAPKs influences the expression of drug-metabolizing enzymes in primary human hepatocytes. *Gen. Physiol. Biophys.* 28 316–320. 10.4149/gpb_2009_03_316 20037198

[B4] Beger R. D. Dunn W. Schmidt M. A. Gross S. S. Kirwan J. A. Cascante M. (2016). Metabolomics enables precision medicine:“a white paper, community perspective.”. *Metabolomics* 12:149. 10.1007/s11306-016-1094-6 27642271 PMC5009152

[B5] Bradley R. M. Marvyn P. M. Henao J. J. A. Mardian E. B. George S. Aucoin M. G. (2015). Acylglycerophosphate acyltransferase 4 (AGPAT4) is a mitochondrial lysophosphatidic acid acyltransferase that regulates brain phosphatidylcholine, phosphatidylethanolamine, and phosphatidylinositol levels. *Biochim. Biophys. Acta* 1851 1566–1576. 10.1016/j.bbalip.2015.09.005 26417903

[B6] De Chiara G. Marcocci M. E. Torcia M. Lucibello M. Rosini P. Bonini P. (2006). Bcl-2 phosphorylation by p38 MAPK Identification of target sites and biologic consequences. *J. Biol. Chem.* 281 21353–21361. 10.1074/jbc.M511052200 16714293

[B7] Dixit A. K. Dey R. Suresh A. Chaudhuri S. Panda A. K. Mitra A. (2014). The prevalence of dyslipidemia in patients with diabetes mellitus of ayurveda Hospital. *J. Diabetes Metab. Disord.* 13:58. 10.1186/2251-6581-13-58 24918095 PMC4051117

[B8] Grab L. T. Kearns M. W. Morris A. J. Daniel L. W. (2004). Differential role for phospholipase D1 and phospholipase D2 in 12-O-tetradecanoyl-13-phorbol acetate-stimulated MAPK activation, Cox-2 and IL-8 expression. *Biochim. Biophys. Acta* 1636 29–39. 10.1016/j.bbalip.2003.12.002 14984736

[B9] Gregory K. E. Bird S. S. Gross V. S. Marur V. R. Lazarev A. V. Walker W. A. (2013). Method development for fecal lipidomics profiling. *Anal. Chem.* 85 1114–1123. 10.1021/ac303011k 23210743 PMC3928122

[B10] Guo C. Yang R.-J. Jang K. Zhou X. Liu Y. (2017). Protective effects of pretreatment with quercetin against lipopolysaccharide-induced apoptosis and the inhibition of osteoblast differentiation via the mapk and wnt/β-catenin pathways in MC3T3-E1 cells. *Cell. Physiol. Biochem.* 43 1547–1561. 10.1159/000481978 29035884

[B11] Huan T. Forsberg E. M. Rinehart D. Johnson C. H. Ivanisevic J. Benton H. P. (2017). Systems biology guided by XCMS Online metabolomics. *Nat. Methods* 14 461–462. 10.1038/nmeth.4260 28448069 PMC5933448

[B12] Huang T. Mi H. Lin C. Zhao L. Zhong L. L. D. Liu F. (2017). MOST: most-similar ligand based approach to target prediction. *BMC Bioinformatics* 18:165. 10.1186/s12859-017-1586-z 28284192 PMC5346209

[B13] Huang T. Ning Z. Hu D. Zhang M. Zhao L. Lin C. (2018). Uncovering the mechanisms of Chinese Herbal Medicine (MaZiRenWan) for functional constipation by focused network pharmacology approach. *Front. Pharmacol.* 9:270. 10.3389/fphar.2018.00270 29632490 PMC5879454

[B14] Jiang C. Wang Q. Wei Y. Yao N. Wu Z. Ma Y. (2015). Cholesterol-lowering effects and potential mechanisms of different polar extracts from *Cyclocarya paliurus* leave in hyperlipidemic mice. *J. Ethnopharmacol.* 176 17–26. 10.1016/j.jep.2015.10.006 26477373

[B15] Jiang C. Yao N. Wang Q. Zhang J. Sun Y. Xiao N. (2014). *Cyclocarya paliurus* extract modulates adipokine expression and improves insulin sensitivity by inhibition of inflammation in mice. *J. Ethnopharmacol.* 153 344–351. 10.1016/j.jep.2014.02.003 24530856

[B16] Jung U. J. Choi M.-S. (2014). Obesity and its metabolic complications: the role of adipokines and the relationship between obesity, inflammation, insulin resistance, dyslipidemia and nonalcoholic fatty liver disease. *Int. J. Mol. Sci.* 15 6184–6223. 10.3390/ijms15046184 24733068 PMC4013623

[B17] Kasumov T. Li L. Li M. Gulshan K. Kirwan J. P. Liu X. (2015). Ceramide as a mediator of non-alcoholic Fatty liver disease and associated atherosclerosis. *PLoS One* 10:e0126910. 10.1371/journal.pone.0126910 25993337 PMC4439060

[B18] Kurihara H. Asami S. Shibata H. Fukami H. Tanaka T. (2003). Hypolipemic Effect of *Cyclocarya paliurus* (Batal) Iljinskaja in lipid-loaded mice. *Biol. Pharm. Bull.* 26 383–385. 10.1248/bpb.26.383 12612454

[B19] Li S. Fan T.-P. Jia W. Lu A. Zhang W. (2014). Network pharmacology in traditional chinese medicine. *Evid. Based Complement. Altern. Med.* 2014:138460. 10.1155/2014/138460 24707305 PMC3953584

[B20] Lin Z. Wu Z.-F. Jiang C.-H. Zhang Q.-W. Ouyang S. Che C.-T. (2016). The chloroform extract of *Cyclocarya paliurus* attenuates high-fat diet induced non-alcoholic hepatic steatosis in Sprague Dawley rats. *Phytomedicine* 23 1475–1483. 10.1016/j.phymed.2016.08.003 27765368

[B21] Ling A. V. Gearing M. E. Semova I. Shin D.-J. Clements R. Lai Z. W. (2017). FoxO1 is required for most of the metabolic and hormonal perturbations produced by hepatic insulin receptor deletion in male mice. *Endocrinology* 159 1253–1263. 10.1210/en.2017-00870 29300910 PMC5802805

[B22] Ma Y. Jiang C. Yao N. Li Y. Wang Q. Fang S. (2015). Antihyperlipidemic effect of *Cyclocarya paliurus* (Batal.) Iljinskaja extract and inhibition of apolipoprotein B48 overproduction in hyperlipidemic mice. *J. Ethnopharmacol.* 166 286–296. 10.1016/j.jep.2015.03.030 25794806

[B23] Niebergall L. J. Jacobs R. L. Chaba T. Vance D. E. (2011). Phosphatidylcholine protects against steatosis in mice but not non-alcoholic steatohepatitis. *Biochim. Biophys. Acta* 1811 1177–1185. 10.1016/j.bbalip.2011.06.021 21745592

[B24] Parhofer K. G. (2015). Interaction between glucose and lipid metabolism: more than diabetic dyslipidemia. *Diabetes Metab. J.* 39 353–362. 10.4093/dmj.2015.39.5.353 26566492 PMC4641964

[B25] Payne F. Lim K. Girousse A. Brown R. J. Kory N. Robbins A. (2014). Mutations disrupting the Kennedy phosphatidylcholine pathway in humans with congenital lipodystrophy and fatty liver disease. *Proc. Natl. Acad. Sci. U.S.A.* 111 8901–8906. 10.1073/pnas.1408523111 24889630 PMC4066527

[B26] Perry R. J. Samuel V. T. Petersen K. F. Shulman G. I. (2014). The role of hepatic lipids in hepatic insulin resistance and type 2 diabetes. *Nature* 510 84–91. 10.1038/nature13478 24899308 PMC4489847

[B27] Rahmani M. Aust M. M. Attkisson E. Williams D. C. J. Ferreira-Gonzalez A. Grant S. (2013). Dual inhibition of Bcl-2 and Bcl-xL strikingly enhances PI3K inhibition-induced apoptosis in human myeloid leukemia cells through a GSK3- and Bim-dependent mechanism. *Cancer Res.* 73 1340–1351. 10.1158/0008-5472.CAN-12-1365 23243017 PMC3578060

[B28] Ramachandran V. Saravanan R. (2015). Glucose uptake through translocation and activation of GLUT4 in PI3K/Akt signaling pathway by asiatic acid in diabetic rats. *Hum. Exp. Toxicol.* 34 884–893. 10.1177/0960327114561663 26286522

[B29] Richhariya A. Fox K. M. Punekar R. S. Gandra S. R. Fisher M. D. Cziraky M. J. (2015). Gender Differences in Recurrent Cardiovascular Events Among High-risk Patients With Hyperlipidemia. *Circ. Cardiovasc. Qual. Outcomes* 8:A307 10.1161/circoutcomes.8.suppl_2.307

[B30] Rosenson R. S. Brewer H. B. Jr. Ansell B. J. Barter P. Chapman M. J. Heinecke J. W. (2016). Dysfunctional HDL and atherosclerotic cardiovascular disease. *Nat. Rev. Cardiol.* 13 48–60. 10.1038/nrcardio.2015.124 26323267 PMC6245940

[B31] Sattar N. Preiss D. Robinson J. G. Djedjos C. S. Elliott M. Somaratne R. (2016). Lipid-lowering efficacy of the PCSK9 inhibitor evolocumab (AMG 145) in patients with type 2 diabetes: a meta-analysis of individual patient data. *Lancet Diabetes Endocrinol.* 4 403–410. 10.1016/S2213-8587(16)00003-6 26868195

[B32] Spence J. D. Urquhart B. L. Bang H. (2016). Effect of renal impairment on atherosclerosis: only partially mediated by homocysteine. *Nephrol. Dial. Transplant.* 31 937–944. 10.1093/ndt/gfv380 26567910 PMC4876968

[B33] Stern C. Castera L. (2017). Non-invasive diagnosis of hepatic steatosis. *Hepatol. Int.* 11 70–78. 10.1007/s12072-016-9772-z 27783208

[B34] Sugiyama M. Banno R. Mizoguchi A. Tominaga T. Tsunekawa T. Onoue T. (2017). PTP1B deficiency improves hypothalamic insulin sensitivity resulting in the attenuation of AgRP mRNA expression under high-fat diet conditions. *Biochem. Biophys. Res. Commun.* 488 116–121. 10.1016/j.bbrc.2017.05.019 28479249

[B35] Szczepaniak L. S. Nurenberg P. Leonard D. Browning J. D. Reingold J. S. Grundy S. (2005). Magnetic resonance spectroscopy to measure hepatic triglyceride content: prevalence of hepatic steatosis in the general population. *Am. J. Physiol. Metab.* 288 E462–E468. 10.1152/ajpendo.00064.2004 15339742

[B36] Szklarczyk D. Franceschini A. Wyder S. Forslund K. Heller D. Huerta-Cepas J. (2014). STRING v10: protein–protein interaction networks, integrated over the tree of life. *Nucleic Acids Res.* 43 D447–D452. 10.1093/nar/gku1003 25352553 PMC4383874

[B37] Wang Q. Jiang C. Fang S. Wang J. Ji Y. Shang X. (2013). Antihyperglycemic, antihyperlipidemic and antioxidant effects of ethanol and aqueous extracts of *Cyclocarya paliurus* leaves in type 2 diabetic rats. *J. Ethnopharmacol.* 150 1119–1127. 10.1016/j.jep.2013.10.040 24184190

[B38] Wang X. Zhang A. Sun H. Han Y. (2017). “Integrated serum pharmacochemistry of TCM and metabolomics strategies for innovative drug discovery,” in *Serum Pharmacochemistry of Traditional Chinese Medicine*, eds Wang X. Zhang A. Sun H. (New York, NY: Elsevier), 15–21.

[B39] Wang Z. Xie J. Yang Y. Zhang F. Wang S. Wu T. (2017). Sulfated *Cyclocarya paliurus* polysaccharides markedly attenuates inflammation and oxidative damage in lipopolysaccharide-treated macrophage cells and mice. *Sci. Rep.* 7:40402. 10.1038/srep40402 28094275 PMC5240341

[B40] Wouters K. van Gorp P. J. Bieghs V. Gijbels M. J. Duimel H. Lütjohann D. (2008). Dietary cholesterol, rather than liver steatosis, leads to hepatic inflammation in hyperlipidemic mouse models of nonalcoholic steatohepatitis. *Hepatology* 48 474–486. 10.1002/hep.22363 18666236

[B41] Wu Z. Gao T. Zhong R. Lin Z. Jiang C. Ouyang S. (2017). Antihyperlipidaemic effect of triterpenic acid-enriched fraction from *Cyclocarya paliurus* leaves in hyperlipidaemic rats. *Pharm. Biol.* 55 712–721. 10.1080/13880209.2016.1267231 28140736 PMC6130609

[B42] Xia J. Sinelnikov I. V. Han B. Wishart D. S. (2015). MetaboAnalyst 3.0—making metabolomics more meaningful. *Nucleic Acids Res.* 43 W251–W257. 10.1093/nar/gkv380 25897128 PMC4489235

[B43] Xiao H. Wen B. Ning Z. Zhai L. Liao C. Lin C. (2017). *Cyclocarya paliurus* tea leaves enhances pancreatic β cell preservation through inhibition of apoptosis. *Sci. Rep.* 7:9155. 10.1038/s41598-017-09641-z 28831132 PMC5567240

[B44] Xu G. Yoshitomi H. Sun W. Guo X. Wu L. Guo X. (2017). *Cyclocarya paliurus* (Batal.) Ijinskaja aqueous extract (CPAE) ameliorates obesity by improving insulin signaling in the hypothalamus of a metabolic syndrome rat model. *Evid. Based Complement. Altern. Med.* 2017:4602153. 10.1155/2017/4602153 28684967 PMC5480046

[B45] Yan G. Wang X. Zhang A. Shi H. Zhou Y. Sun H. (2017). “Serum pharmacochemistry of TCM for determining the active ingredients of shuanghuanglian formulae,” in *Serum Pharmacochemistry of Traditional Chinese Medicine*, eds Wang X. Zhang A. Sun H. (New York, NY: Elsevier), 155–169. 10.1016/B978-0-12-811147-5.00011-X

[B46] Yang Z.-W. Ouyang K.-H. Zhao J. Chen H. Xiong L. Wang W.-J. (2016). Structural characterization and hypolipidemic effect of *Cyclocarya paliurus* polysaccharide in rat. *Int. J. Biol. Macromol.* 91 1073–1080. 10.1016/j.ijbiomac.2016.06.063 27343704

[B47] Yao X. Lin Z. Jiang C. Gao M. Wang Q. Yao N. (2015). *Cyclocarya paliurus* prevents high fat diet induced hyperlipidemia and obesity in Sprague–Dawley rats. *Can. J. Physiol. Pharmacol.* 93 677–686. 10.1139/cjpp-2014-0477 26203820

[B48] Yoshitomi H. Tsuru R. Li L. Zhou J. Kudo M. Liu T. (2017). *Cyclocarya paliurus* extract activates insulin signaling via Sirtuin1 in C2C12 myotubes and decreases blood glucose level in mice with impaired insulin secretion. *PLoS One* 12:e0183988. 10.1371/journal.pone.0183988 28859155 PMC5578601

[B49] Zafrir B. Jain M. (2014). Lipid-lowering therapies, glucose control and incident diabetes: evidence, mechanisms and clinical implications. *Cardiovasc. Drugs Ther.* 28 361–377. 10.1007/s10557-014-6534-9 24952127

[B50] Zhang P. Xu D. Wang S. Fu H. Wang K. Zou Y. (2011). Inhibition of aldehyde dehydrogenase 2 activity enhances antimycin-induced rat cardiomyocytes apoptosis through activation of MAPK signaling pathway. *Biomed. Pharmacother.* 65 590–593. 10.1016/j.biopha.2009.12.001 21123025

[B51] Zhao J. O’Donnell V. B. Balzar S. Croix C. M. S. Trudeau J. B. Wenzel S. E. (2011). 15-Lipoxygenase 1 interacts with phosphatidylethanolamine-binding protein to regulate MAPK signaling in human airway epithelial cells. *Proc. Natl. Acad. Sci. U.S.A.* 108 14246–14251. 10.1073/pnas.1018075108 21831839 PMC3161579

[B52] Zhao X. Zheng X. Fan T.-P. Li Z. Zhang Y. Zheng J. (2015). A novel drug discovery strategy inspired by traditional medicine philosophies. *Science* 347 S38–S40.

