# Supplementary material for: Cyclocarya paliurus Leaves Tea Improves Dyslipidemia in Diabetic Mice: A Lipidomics-Based Network Pharmacology Study
Source: Front Pharmacol. 2018 Aug 28;9:973. doi: 10.3389/fphar.2018.00973 (PMC6121037; doi:10.3389/fphar.2018.00973)
Supplement: Supplementary file 2 [file Table_2.DOCX]

Supplement Table.S2 The predicted targets of CP components and predicted targets linked lipid targets through protein-protein interactions

| Predicted target | Lipids target | Astragalin | Quercetin | Kaempferol | Neochlorogenic acid | Chlorogenic acid | 4-hydroxybenzoic acid | Gallic acid | Quadranoside IV | Asiatic acid |
| --- | --- | --- | --- | --- | --- | --- | --- | --- | --- | --- |
| ABCC1 | STT3B | √ | √ | √ |  |  |  |  |  |  |
| ALOX12 | PLA2G12A | √ | √ | √ |  |  |  |  |  |  |
| ALOX12 | PLA2G3 | √ | √ | √ |  |  |  |  |  |  |
| ALOX15 | PLA2G12A | √ | √ | √ |  |  |  |  |  |  |
| ALOX15 | PLA2G3 | √ | √ | √ |  |  |  |  |  |  |
| ALOX15 | PLA2G4D | √ | √ | √ |  |  |  |  |  |  |
| APP | PIK3CA | √ | √ | √ | √ | √ |  |  |  |  |
| APP | PLD2 | √ | √ | √ | √ | √ |  |  |  |  |
| BCL2 | PIK3C3 | √ | √ | √ |  |  |  | √ |  |  |
| BCL2 | PIK3CA | √ | √ | √ |  |  |  | √ |  |  |
| BCL2 | PIK3CB | √ | √ | √ |  |  |  | √ |  |  |
| BCL2 | PLA2G1B | √ | √ | √ |  |  |  | √ |  |  |
| BCL2 | PLD2 | √ | √ | √ |  |  |  | √ |  |  |
| CA9 | PTEN | √ | √ | √ | √ | √ |  | √ |  |  |
| CYP2C9 | PLA2G12A | √ | √ | √ |  |  |  |  |  |  |
| CYP2C9 | PLA2G3 | √ | √ | √ |  |  |  |  |  |  |
| ACE | PIK3C2A |  |  |  |  |  | √ |  |  |  |
| CES1 | LIPA |  |  |  |  |  | √ |  |  |  |
| CES1 | LRAT |  |  |  |  |  | √ |  |  |  |
| FYN | PIK3CA |  |  |  | √ | √ |  |  |  |  |
| FYN | PIK3CB |  |  |  | √ | √ |  |  |  |  |
| FYN | PIP5K1C |  |  |  | √ | √ |  |  |  |  |
| FYN | PLD2 |  |  |  | √ | √ |  |  |  |  |
| PSMB8 | PNLIP |  |  |  |  |  |  | √ |  |  |
| PTPN1 | PIK3CA |  |  |  |  |  |  |  | √ | √ |
| PTPN1 | PIK3CG |  |  |  |  |  |  |  | √ | √ |
| PTPN1 | PLD2 |  |  |  |  |  |  |  | √ | √ |
| PTPN2 | PIK3CB |  |  |  |  |  |  |  | √ | √ |

Supplement Table. S3 The components-predicted target-lipids target network of CP.

| Flavonoids  Quercetin; Kaempferol | Association with  Diabetic dyslipidemia | Pharmacology experiment | Predicted targets linked lipids targets | Association with  Diabetic dyslipidemia | Experimental validation of protein-protein interactions |
| --- | --- | --- | --- | --- | --- |
| Predicted targets |  |  |  |  |  |
| ABCC1 | (Behdad et al., 2017) | (Chen et al., 2018) | STT3B |  |  |
| ALOX12 | (Burdon et al., 2010) | (Kizawa et al., 2017) | PLA2G12A  PLA2G3 |  | √ |
| ALOX15 | (Zhao et al., 2011) | (Luiz da Silva et al., 1998) | PLA2G3  PLA2G4D |  |  |
| APP | (Hamilton et al., 2014) | (Martin-Aragon et al., 2016) | PIK3CA | (Ramachandran and Saravanan, 2015) |  |
|  |  |  | PLD2 |  |  |
| BCL2 | (Tomita, 2016) | (Chander et al., 2014) | PIK3CA  PIK3CB  PIK3C3 |  |  |
|  |  |  | PLA2G1B |  |  |
|  |  |  | PLD2 |  |  |
| CA9 |  |  | PTEN | (Birnbaum et al., 2014) |  |
| CYP2CP | (Klen et al., 2014) | (Bedada and Neerati, 2018) | PLA2G12A  PLA2G3 |  |  |

| Organic acids | Predicted targets | Association with diabetic dyslipidemia | Pharmacology experiment | Predicted targets linked lipids targets | Association with diabetic dyslipidemia | Experimental validation of protein-protein interactions |
| --- | --- | --- | --- | --- | --- | --- |
| Chlorogenic acid and neochlorogenic acid | APP | √ |  | PIK3CA | √ |  |
|  | APP | √ |  | PLD2 | √ |  |
|  | CA2 | (Biswas and Kumar, 2012) |  | PTEN | √ |  |
|  | FYN | (Lv et al., 2016) | (Kang et al., 2009) | PIK3CA  PIK3CB | √ | √ |
|  |  |  |  | PIP5K1C |  | √ |
|  |  |  |  | PLD2 | √ | √ |
| Gallic acid | BCL2 | √ | (Yoon et al., 2013) | PIK3CA  PIK3CB  PIK3C3 | √ | √ |
|  |  |  |  | PLA2G1B | √ |  |
|  |  |  |  | PLD2 | √ |  |
|  | CA2 | √ |  | PTEN | √ |  |
|  | PSMB8 |  |  | PNLIP |  |  |
| 4-Hydrobenzoic acid | ACE | (Settin et al., 2015) |  | PIK3C2A | √ |  |
|  | CES1 | (Xu et al., 2014) |  | LIPA |  |  |
|  | CYP2C9 | √ |  | LRAT |  | √ |
|  | PTPN1 | √ |  | PIK3CA  PIK3CB  PIK3C3 |  | √ |

| Saponins: Quadranoside IV and Asiatic acid | Association with diabetic dyslipidemia | Pharmacology experiment | Predicted targets linked lipids targets | Association with diabetic dyslipidemia | Experimental validation of protein-protein interactions |
| --- | --- | --- | --- | --- | --- |
| Predicted targets |  |  |  |  |  |
| PTPN1 | (Cheyssac et al., 2006) |  | PIK3CA  PIK3CG  PIK3CB | √ |  |
| PTPN1 |  |  | PLD2 | √ |  |

References:

Bedada, S. K., and Neerati, P. (2018). Evaluation of the effect of quercetin treatment on CYP2C9 enzyme activity of diclofenac in healthy human volunteers. *Phytother. Res.* 32, 305–311. doi:10.1002/ptr.5978.

Behdad, N., Kojuri, J., Azarpira, N., Masoomi, A., and Namazi, S. (2017). Association of ABCB1 (C3435T) and ABCC1 (G2012T) polymorphisms with clinical response to atorvastatin in Iranian patients with primary hyperlipidemia. *Iran. Biomed. J.* 21, 120–125. doi:10.18869/acadpub.ibj.21.2.120.

Birnbaum, Y., Nanhwan, M. K., Ling, S., Perez-Polo, J. R., Ye, Y., and Bajaj, M. (2014). PTEN upregulation may explain the development of insulin resistance and type 2 diabetes with high dose statins. *Cardiovasc. drugs Ther.* 28, 447–457. doi:10.1007/s10557-014-6546-5.

Biswas, U. K., and Kumar, A. (2012). Study on the changes of carbonic anhydrase activity in insulin resistance and the effect of methylglyoxal. *J. Pak. Med. Assoc.* 62, 417–421.

Burdon, K. P., Rudock, M. E., Lehtinen, A. B., Langefeld, C. D., Bowden, D. W., Register, T. C., et al. (2010). Human lipoxygenase pathway gene variation and association with markers of subclinical atherosclerosis in the diabetes heart study. *Mediators Inflamm.* 2010.

Chander, K., Vaibhav, K., Ejaz Ahmed, M., Javed, H., Tabassum, R., Khan, A., et al. (2014). Quercetin mitigates lead acetate-induced behavioral and histological alterations via suppression of oxidative stress, Hsp-70, Bak and upregulation of Bcl-2. *Food Chem. Toxicol.* 68, 297–306. doi:10.1016/j.fct.2014.02.012.

Chen, Z., Huang, C., Ma, T., Jiang, L., Tang, L., Shi, T., et al. (2018). Reversal effect of quercetin on multidrug resistance via FZD7/beta-catenin pathway in hepatocellular carcinoma cells. *Phytomedicine* 43, 37–45. doi:10.1016/j.phymed.2018.03.040.

Cheyssac, C., Lecoeur, C., Dechaume, A., Bibi, A., Charpentier, G., Balkau, B., et al. (2006). Analysis of common PTPN1 gene variants in type 2 diabetes, obesity and associated phenotypes in the French population. *BMC Med. Genet.* 7, 44. doi:10.1186/1471-2350-7-44.

Hamilton, D. L., Findlay, J. A., Montagut, G., Meakin, P. J., Bestow, D., Jalicy, S. M., et al. (2014). Altered amyloid precursor protein processing regulates glucose uptake and oxidation in cultured rodent myotubes. *Diabetologia* 57, 1684–1692. doi:10.1007/s00125-014-3269-x.

Kang, N. J., Lee, K. W., Shin, B. J., Jung, S. K., Hwang, M. K., Bode, A. M., et al. (2009). Caffeic acid, a phenolic phytochemical in coffee, directly inhibits Fyn kinase activity and UVB-induced COX-2 expression. *Carcinogenesis* 30, 321–330. doi:10.1093/carcin/bgn282.

Kizawa, K., Fujimori, T., and Kawai, T. (2017). Arachidonate 12-Lipoxygenase Inhibitors Promote S100A3 Citrullination in Cultured SW480 Cells and Isolated Hair Follicles. *Biol. Pharm. Bull.* 40, 516–523. doi:10.1248/bpb.b16-00954.

Klen, J., Dolzan, V., and Janez, A. (2014). CYP2C9, KCNJ11 and ABCC8 polymorphisms and the response to sulphonylurea treatment in type 2 diabetes patients. *Eur. J. Clin. Pharmacol.* 70, 421–428. doi:10.1007/s00228-014-1641-x.

Luiz da Silva, E., Tsushida, T., and Terao, J. (1998). Inhibition of mammalian 15-lipoxygenase-dependent lipid peroxidation in low-density lipoprotein by quercetin and quercetin monoglucosides. *Arch. Biochem. Biophys.* 349, 313–320.

Lv, Z., Hu, M., Ren, X., Fan, M., Zhen, J., Chen, L., et al. (2016). Fyn Mediates High Glucose-Induced Actin Cytoskeleton Reorganization of Podocytes via Promoting ROCK Activation In Vitro. *J. Diabetes Res.* 2016, 5671803. doi:10.1155/2016/5671803.

Martin-Aragon, S., Jimenez-Aliaga, K. L., Benedi, J., and Bermejo-Bescos, P. (2016). Neurohormetic responses of quercetin and rutin in a cell line over-expressing the amyloid precursor protein (APPswe cells). *Phytomedicine* 23, 1285–1294. doi:10.1016/j.phymed.2016.07.007.

Ramachandran, V., and Saravanan, R. (2015). Glucose uptake through translocation and activation of GLUT4 in PI3K/Akt signaling pathway by asiatic acid in diabetic rats. *Hum. Exp. Toxicol.* 34, 884–893. doi:10.1177/0960327114561663.

Settin, A., El-Baz, R., Ismaeel, A., Tolba, W., and Allah, W. A. (2015). Association of ACE and MTHFR genetic polymorphisms with type 2 diabetes mellitus: Susceptibility and complications. *J. Renin. Angiotensin. Aldosterone. Syst.* 16, 838–843. doi:10.1177/1470320313516172.

Tomita, T. (2016). Apoptosis in pancreatic beta-islet cells in Type 2 diabetes. *Bosn. J. basic Med. Sci.* 16, 162–179. doi:10.17305/bjbms.2016.919.

Xu, J., Yin, L., Xu, Y., Li, Y., Zalzala, M., Cheng, G., et al. (2014). Hepatic carboxylesterase 1 is induced by glucose and regulates postprandial glucose levels. *PLoS One* 9, e109663. doi:10.1371/journal.pone.0109663.

Yoon, C.-H., Chung, S.-J., Lee, S.-W., Park, Y.-B., Lee, S.-K., and Park, M.-C. (2013). Gallic acid, a natural polyphenolic acid, induces apoptosis and inhibits proinflammatory gene expressions in rheumatoid arthritis fibroblast-like synoviocytes. *Joint. Bone. Spine* 80, 274–279. doi:10.1016/j.jbspin.2012.08.010.

Zhao, J., O’Donnell, V. B., Balzar, S., Croix, C. M. S., Trudeau, J. B., and Wenzel, S. E. (2011). 15-Lipoxygenase 1 interacts with phosphatidylethanolamine-binding protein to regulate MAPK signaling in human airway epithelial cells. *Proc. Natl. Acad. Sci.* 108, 14246–14251.
