# Supplementary material for: Cyclocarya paliurus Leaves Tea Improves Dyslipidemia in Diabetic Mice: A Lipidomics-Based Network Pharmacology Study
Source: Front Pharmacol. 2018 Aug 28;9:973. doi: 10.3389/fphar.2018.00973 (PMC6121037; doi:10.3389/fphar.2018.00973)
Supplement: Supplementary file 3 [file Data_Sheet_1.PDF]

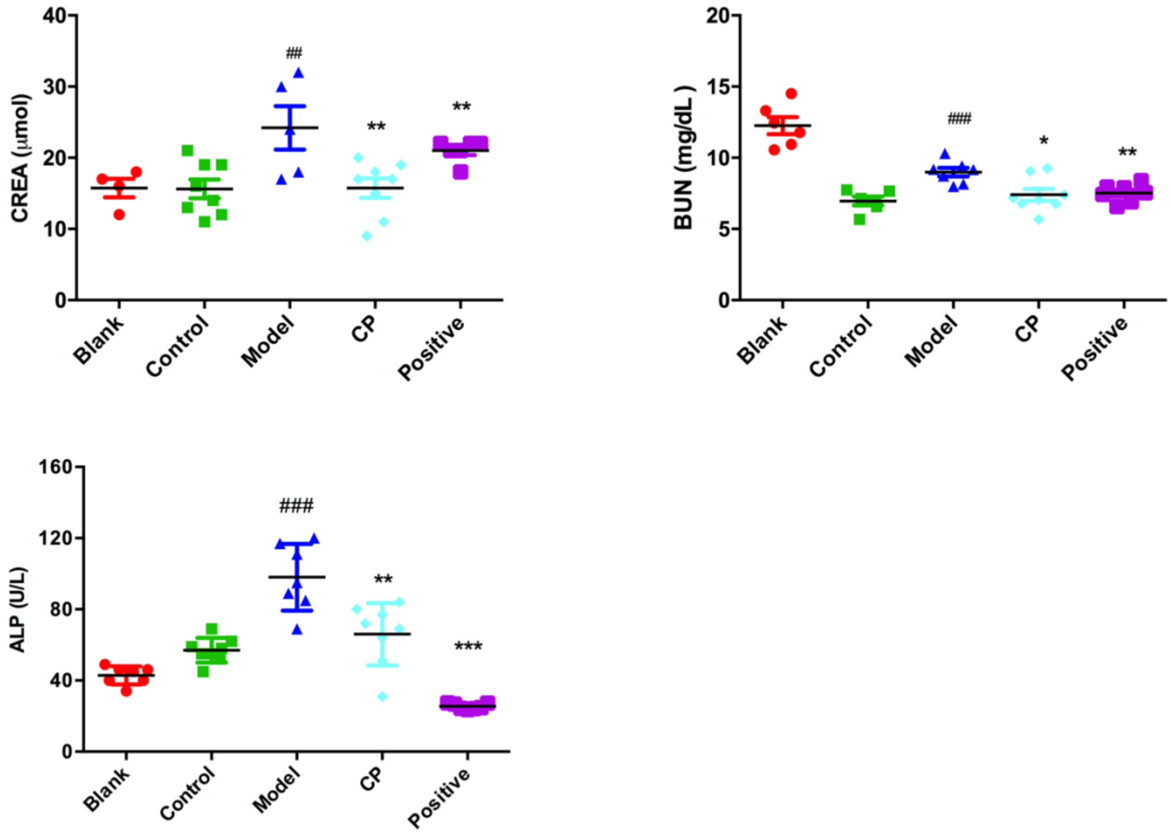

*Supplement Figure. S1 The CP attenuated diabetes complications induced by high fat diet and STZ in mice. Blank: non-diabetic control group; Control: CP-treated non-diabetic control group; Model: STZ and high fat diet induced diabetic model group; CP: CP treated model group; Positive: glibenclamide treated model group. CREA: Creatinine level; BUN: Blood urea nitrogen level; ALP: Alkaline phosphatase level. All data are presented as means + SEM (n=6~8). #p<0.05, ##p<0.01, ###p<0.001 (comparison between control and model group); *p<0.05, ** p<0.01, *** p<0.001 (comparison between CP group, positive group and model group).*

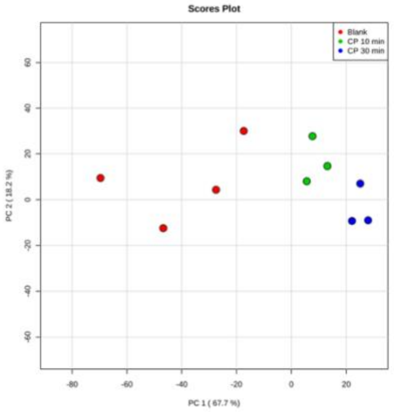

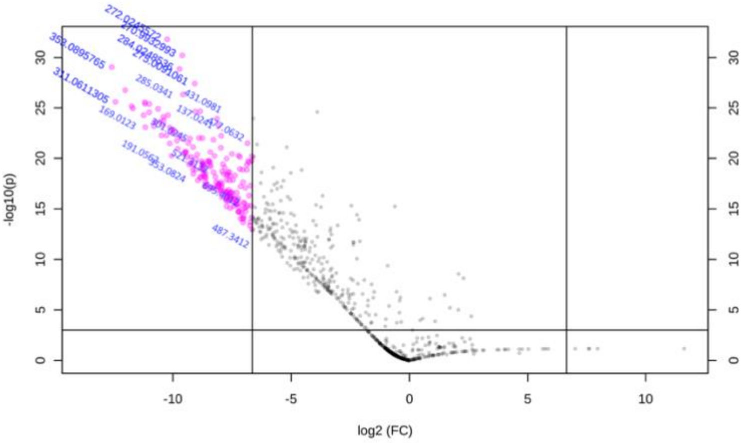

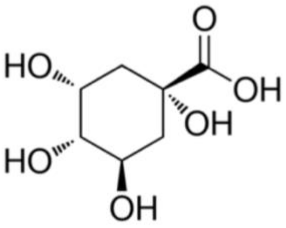

Quinic acid

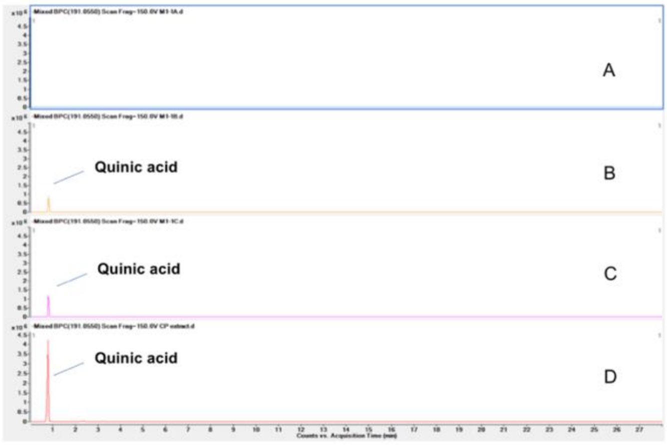

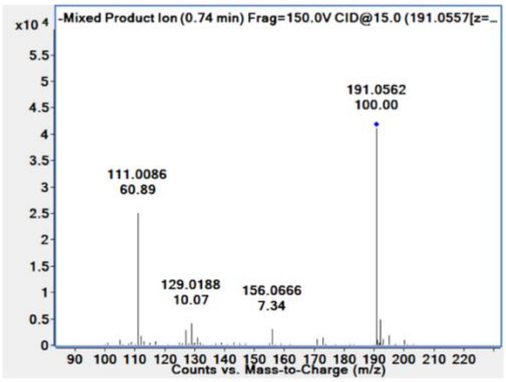

A. Blank plasma
B. Plasma after administration of CP at 10min
C. Plasma after administration of CP extract at 30min
D. CP extract

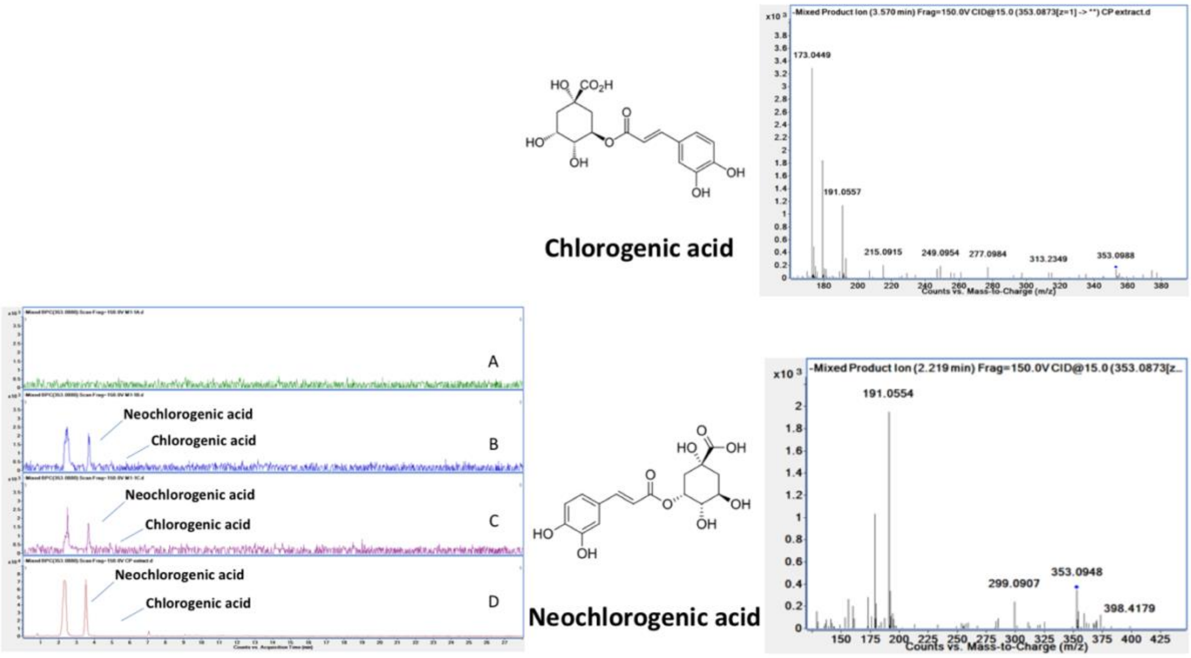

A. Blank plasma
B. Plasma after administration of CP at 10min
C. Plasma after administration of CP extract at 30min
D. CP extract

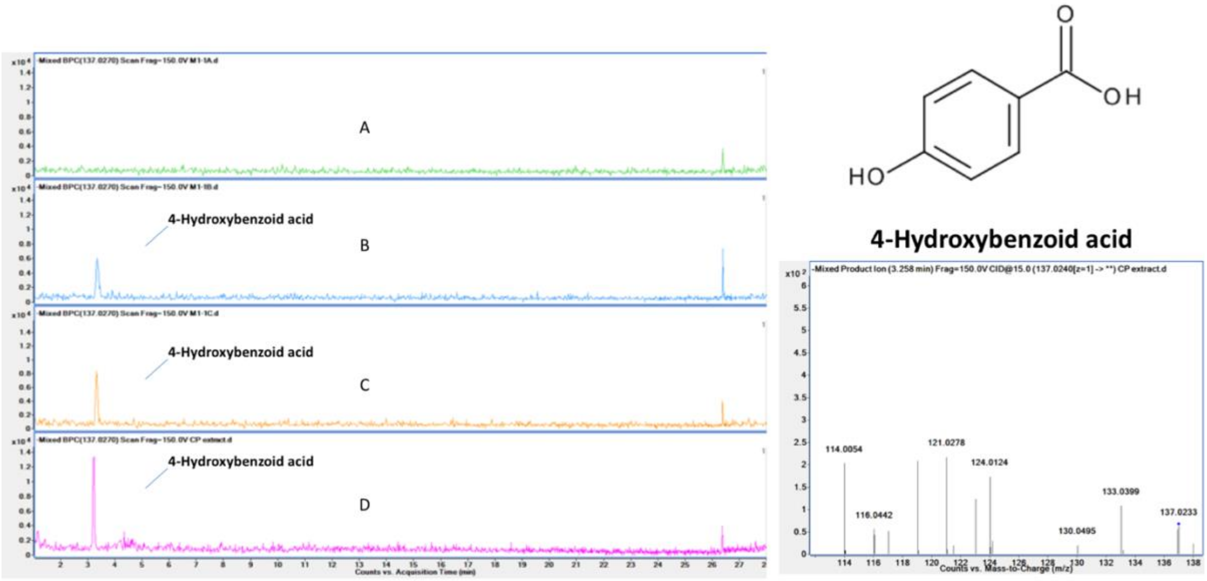

A. Blank plasma
B. Plasma after administration of CP at 10min
C. Plasma after administration of CP extract at 30min
D. CP extract

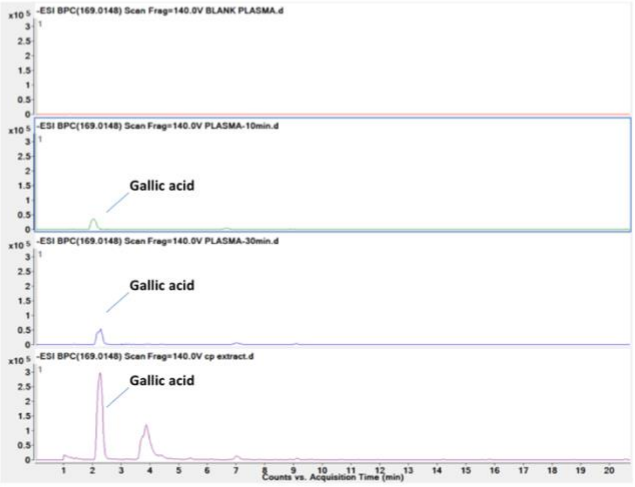

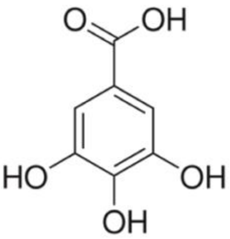

Gallic acid

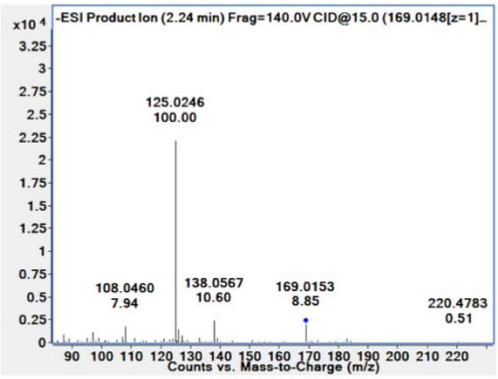

A. Blank plasma **B. Plasma after administration of CP at 10min**
C. Plasma after administration of CP extract at 30min **D. CP extract**

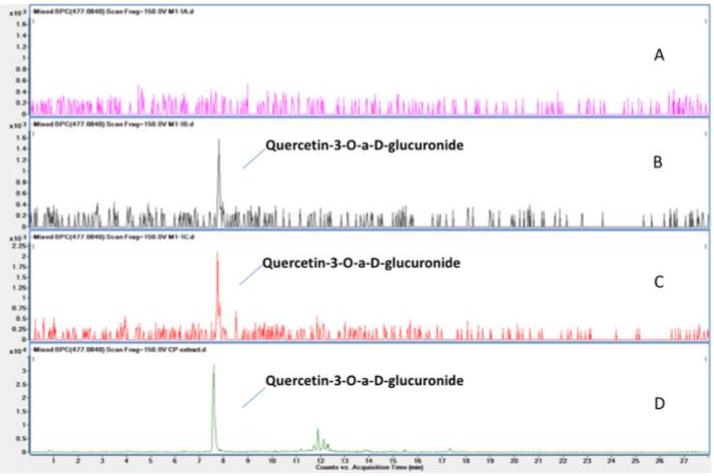

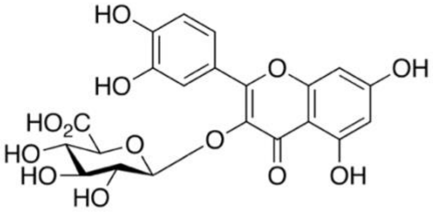

Quercetin-3-O-a-D-glucuronide

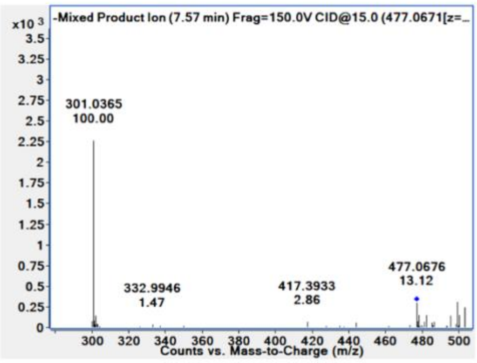

A. Blank plasma **B. Plasma after administration of CP at 10min**
C. Plasma after administration of CP extract at 30min **D. CP extract**

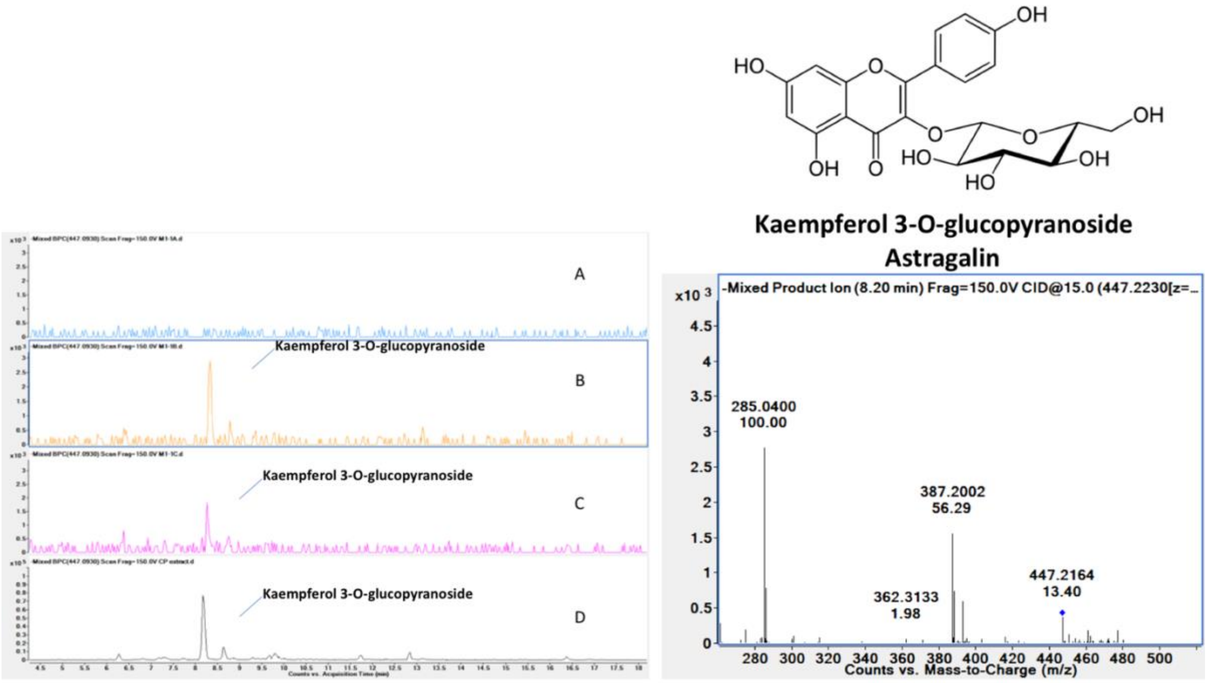

A. Blank plasma
B. Plasma after administration of CP at 10min
C. Plasma after administration of CP extract at 30min
D. CP extract

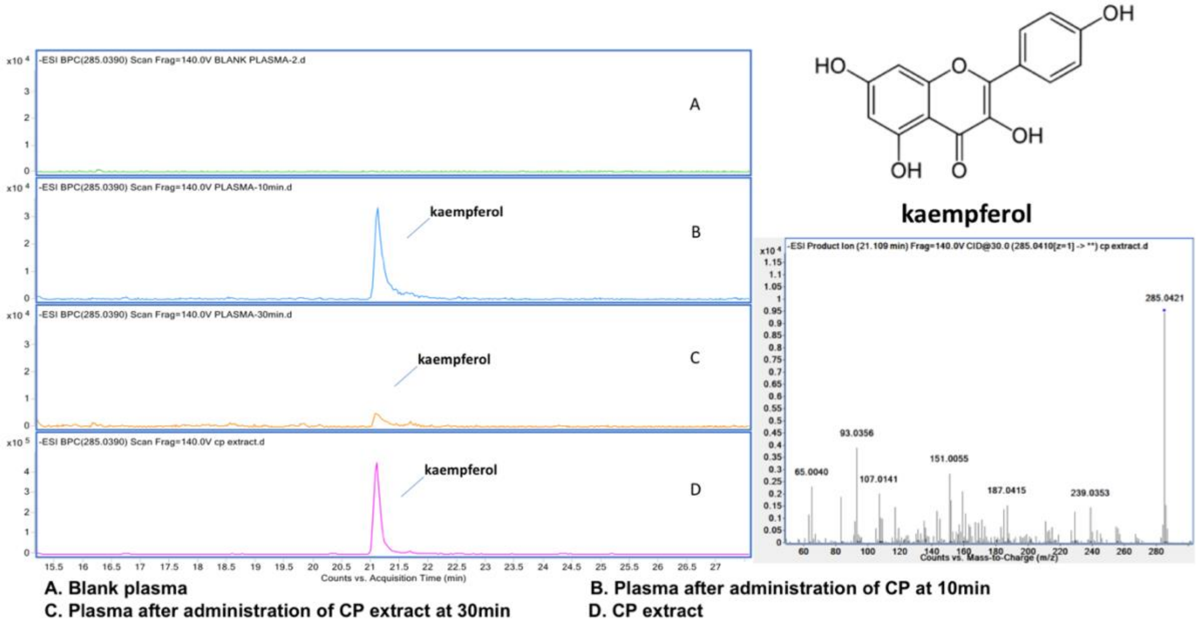

A. Blank plasma
B. Plasma after administration of CP at 10min
C. Plasma after administration of CP extract at 30min
D. CP extract

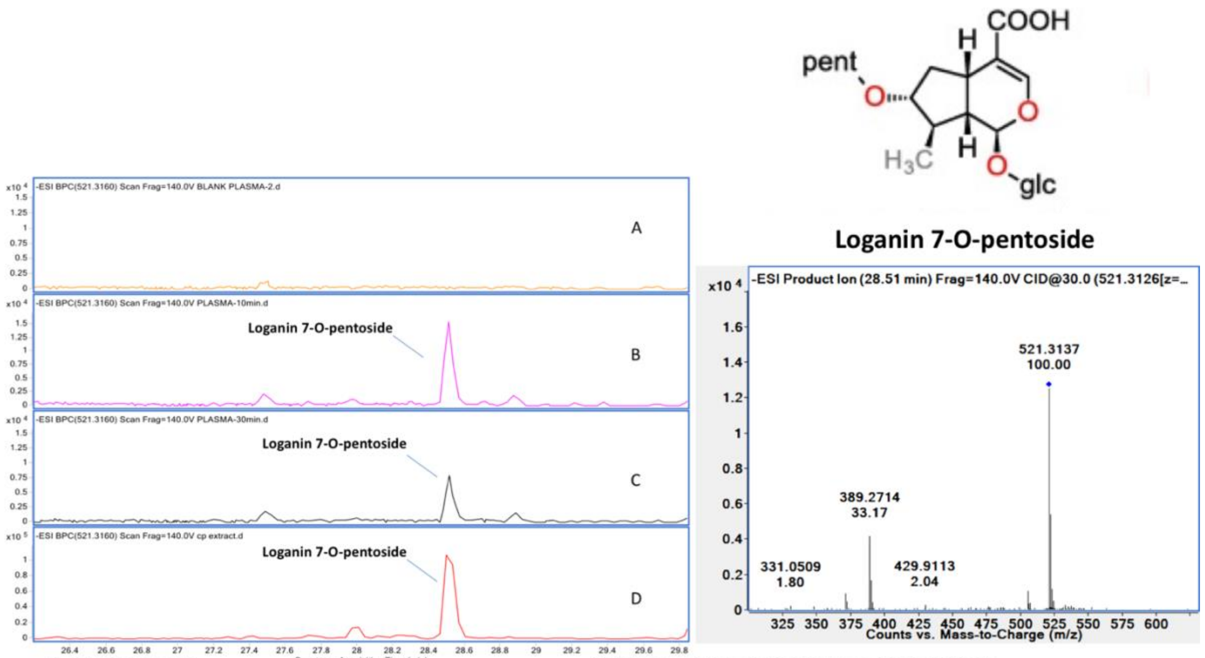

A. Blank plasma
B. Plasma after administration of CP at 10min
C. Plasma after administration of CP extract at 30min
D. CP extract

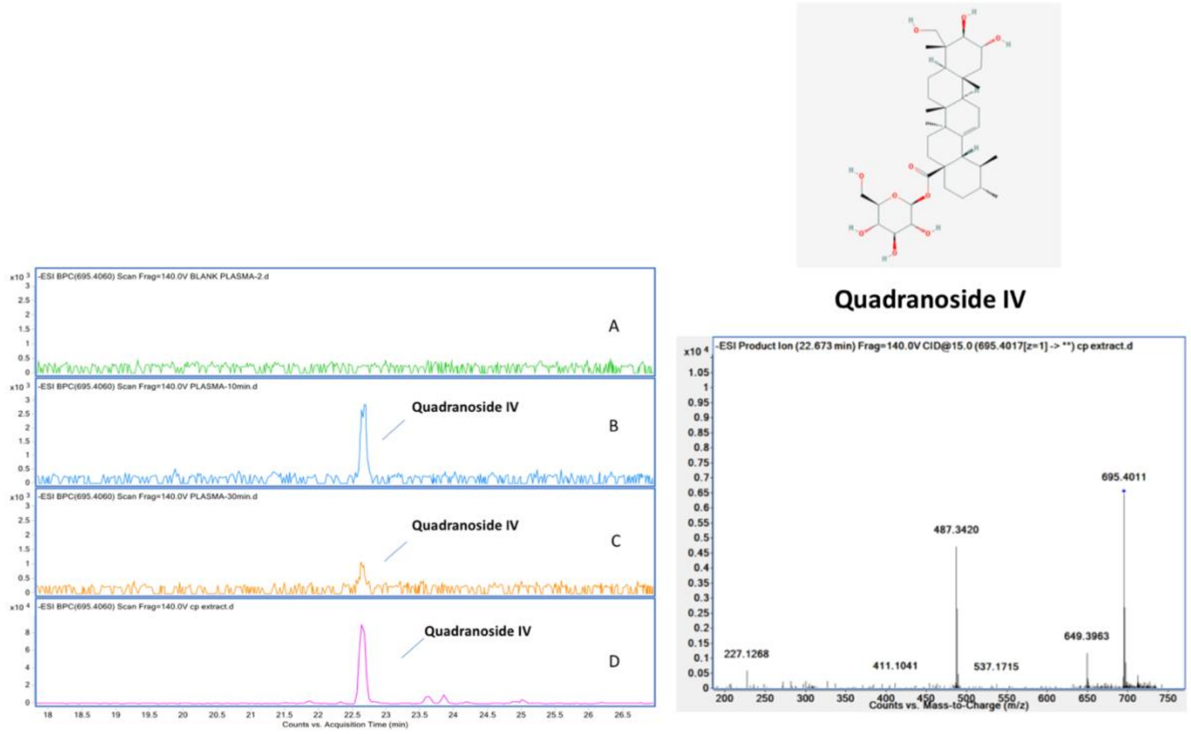

A. Blank plasma
B. Plasma after administration of CP at 10min
C. Plasma after administration of CP extract at 30min
D. CP extract

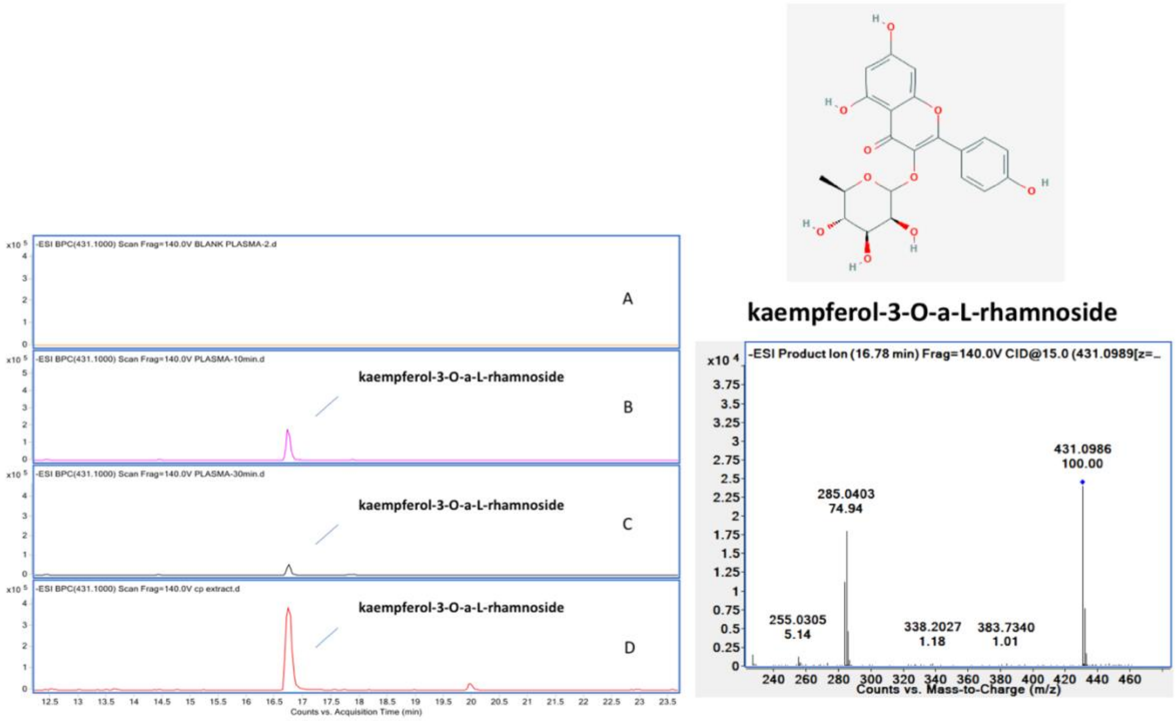

A. Blank plasma
B. Plasma after administration of CP at 10min
C. Plasma after administration of CP extract at 30min
D. CP extract

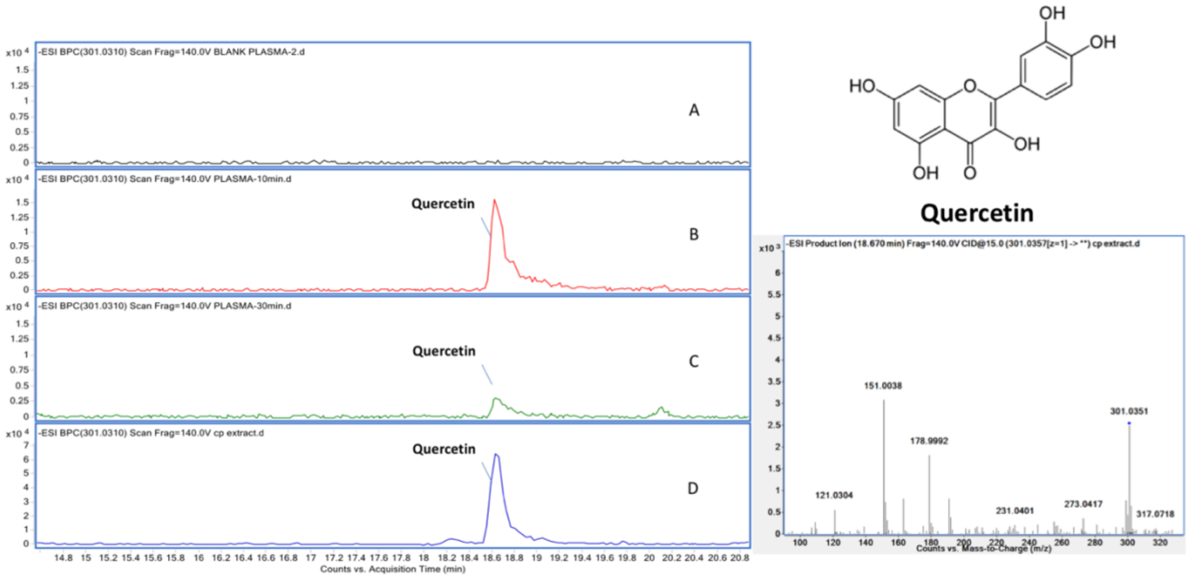

A. Blank plasma
B. Plasma after administration of CP at 10min
C. Plasma after administration of CP extract at 30min
D. CP extract

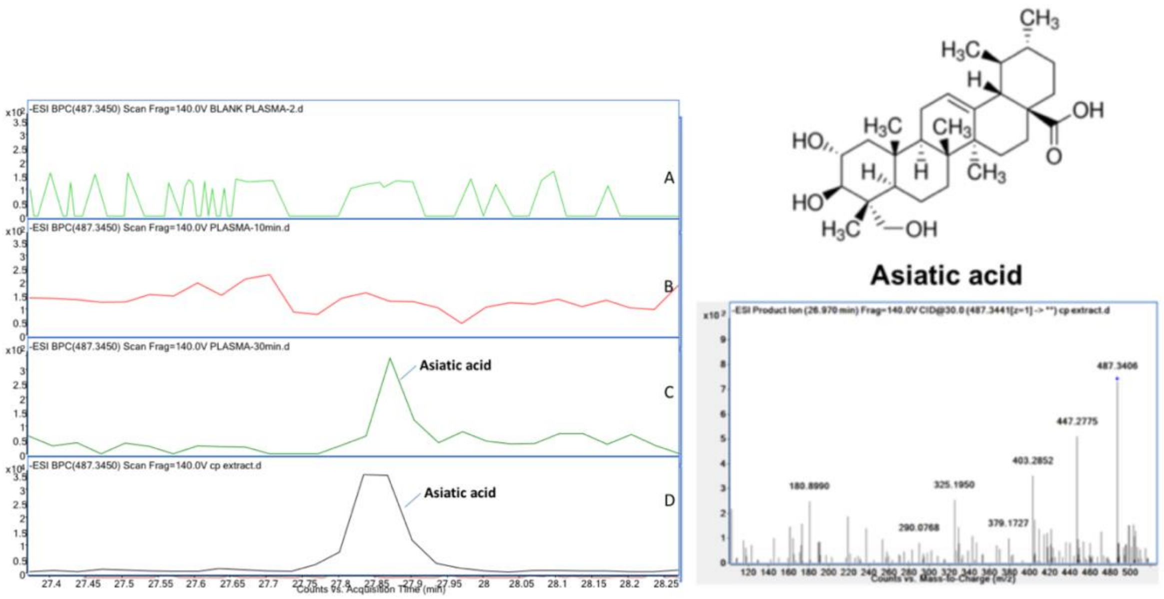

A. Blank plasma
B. Plasma after administration of CP at 10min
C. Plasma after administration of CP extract at 30min
D. CP extract

Supplement Figure. S2 The base peak current (BPC) chromatogram of CP constituents and their associated MS/MS chromatogram detected in bloodstream.

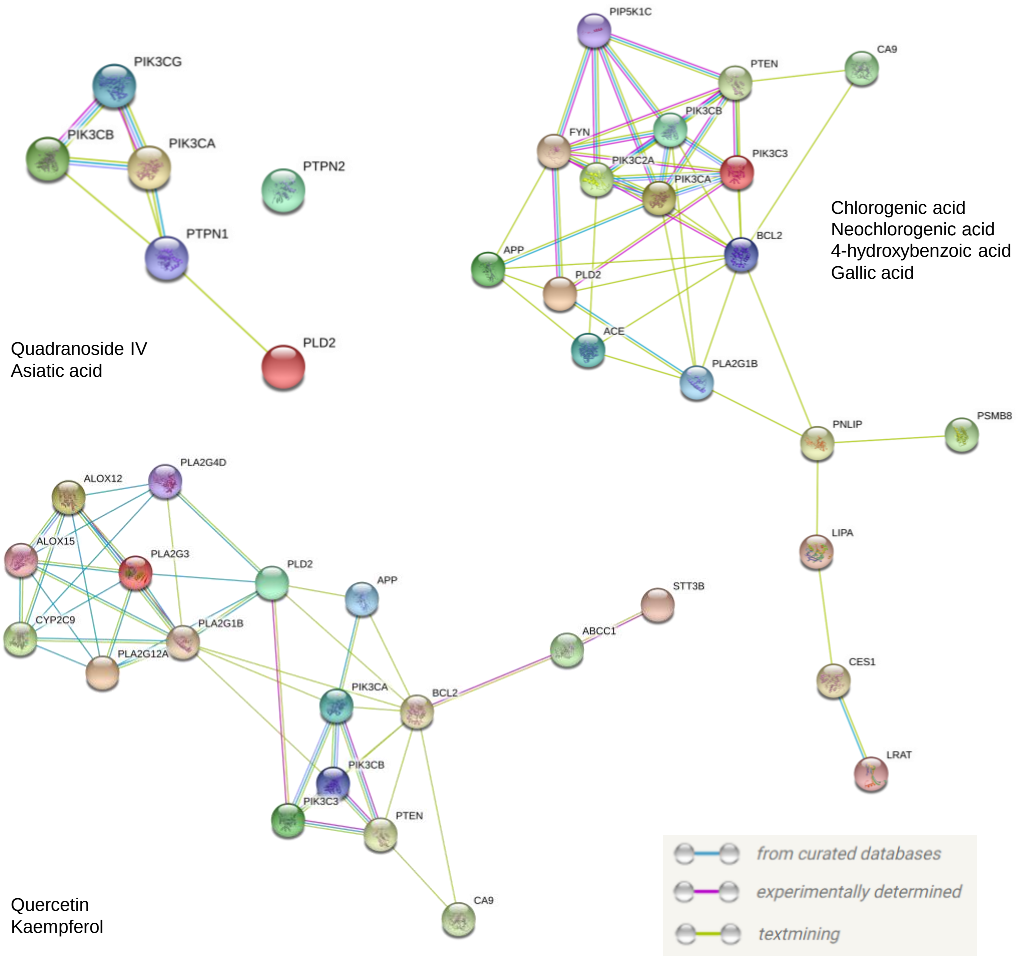

Supplement Figure.S3 The protein-protein interactions networks by STRING. The integrate protein-protein interactions prediction plot of selected lipids and predicted targets of CP by MOST. The PPI networks by different constitutes of CP. The CP constituents were classified by chemical similarity in PPI analysis. All the targets above were either predicted targets or lipids targets. The PPI between predicted targets and lipids targets were explained. The blue line represented the interaction was based from curated databases. The purple line indicated the interactions was determined by experiment. The yellow line indicated the interaction was based on text mining.

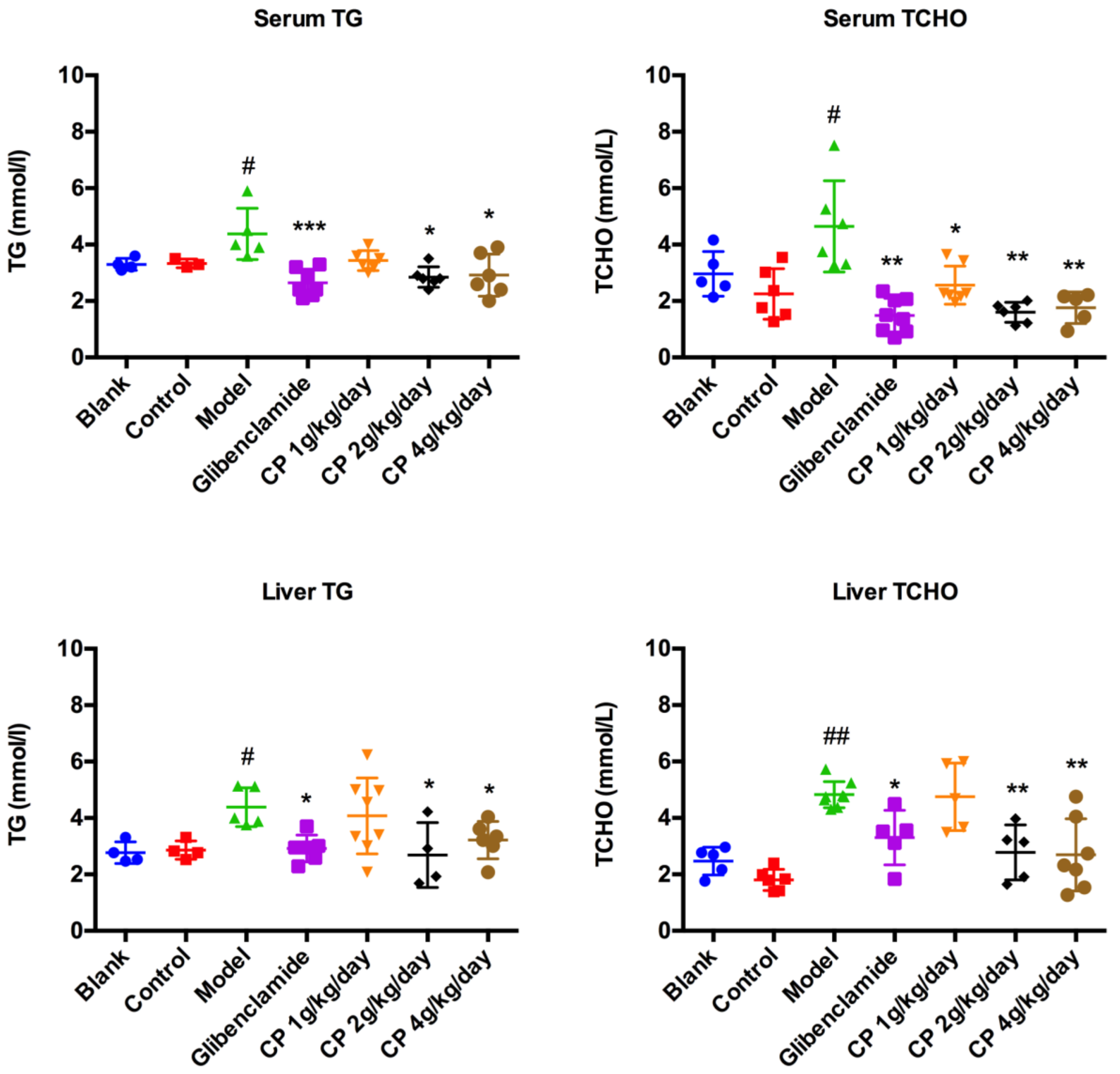

*Supplement Figure S4 TG and T-CHO in serum and liver tissue. All data are presented as means ± SEM (n=6~8). #p<0.05, ##p<0.01, ###p<0.001 (comparison between control and model group); *p<0.05, ** p<0.01, *** p<0.001 (comparison between CP group and model group).*
