# Supplementary material for: Cyclocarya paliurus Leaves Tea Improves Dyslipidemia in Diabetic Mice: A Lipidomics-Based Network Pharmacology Study
Source: Front Pharmacol. 2018 Aug 28;9:973. doi: 10.3389/fphar.2018.00973 (PMC6121037; doi:10.3389/fphar.2018.00973)
Supplement: Supplementary file 4 [file Data_Sheet_2.PDF]

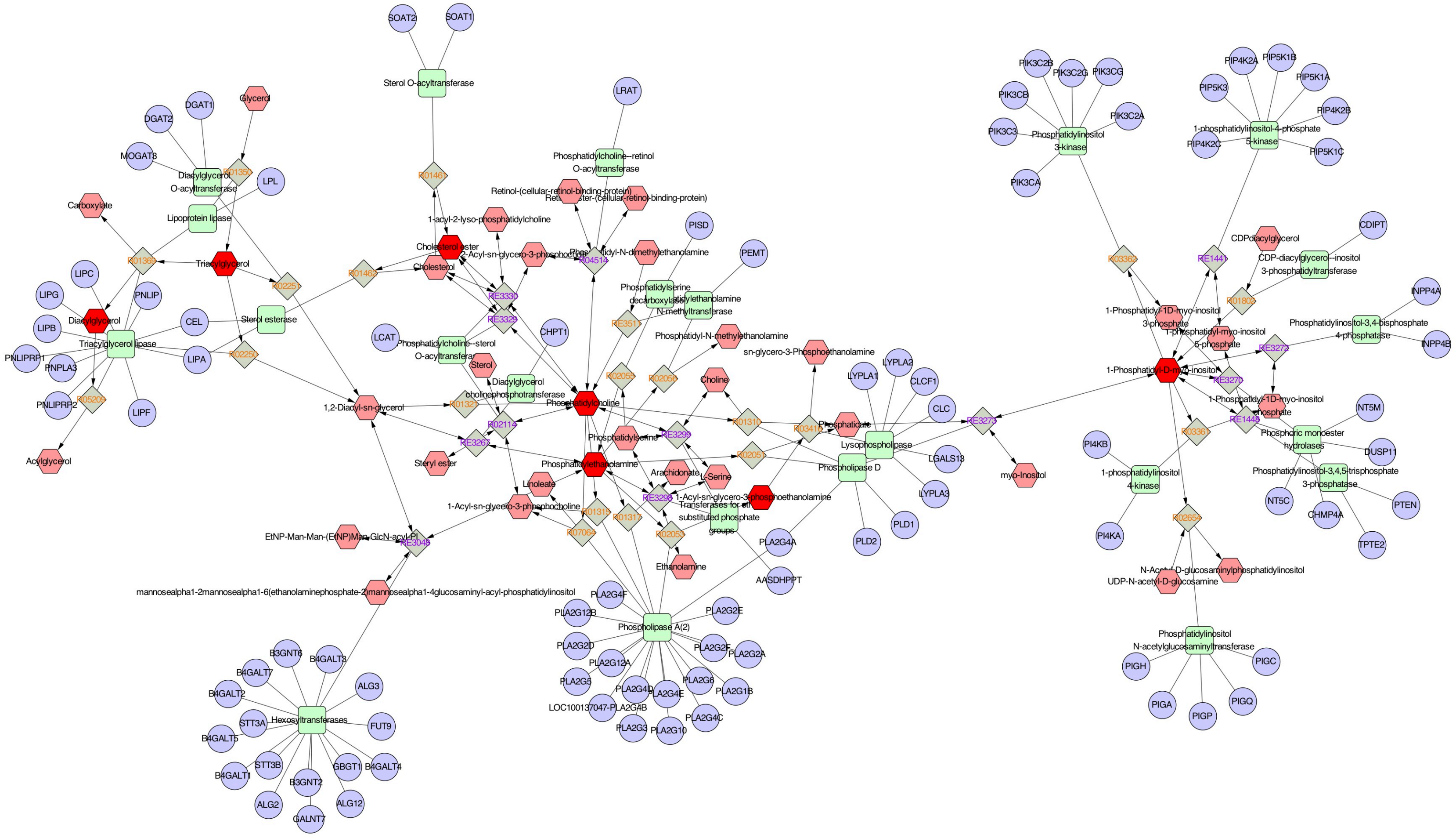

Supplement Figure.S5 The pathway map of associated lipids-reaction-enzyme-gene selected lipids networks. The dark red hexagonal represents detected metabolites, the shallow red hexagonal represents in-direct metabolites. The green square represents protein (enzyme). The blue circle represents genes that coding for the protein.
